# Enactivism in Autism Healthcare Practices: A Scoping Review

**DOI:** 10.1177/00469580261467452

**Published:** 2026-07-13

**Authors:** Johannes Cornelis van Huizen, Jan-Pieter Teunisse, Rosa H. van Doormaal, Antje Orgassa, Wouter G. Staal, Derek W. Strijbos

**Affiliations:** 1 School of Social Studies, HAN University of Applied Sciences, Nijmegen, The Netherlands; 2 Research and Development, Leo Kannerhuis, Arnhem, The Netherlands; 3 Independent researcher, Nijmegen, The Netherlands; 4 Department of Psychiatry, Radboud University Medical Center, Nijmegen, The Netherlands; 5 Leiden Institute for Brain and Cognition (LIBC), Leiden University, Leiden, The Netherlands; 6 University Centre, Karakter Child and Adolescent Psychiatry, Nijmegen, The Netherlands; 7 Faculty of Philosophy, Theology, and Religious Studies, Radboud University, Nijmegen, The Netherlands; 8 Specialist Centre for Developmental Disorders, Dimence Group Mental Health Center, Deventer, The Netherlands

**Keywords:** autopoietic enactivism_**1**_, enactivism_**2**_, self-regulation_**2**_, sense-making_**3**_, autism healthcare practices_**4**_, complexity_**5**_, integration_**6**_

## Abstract

**Introduction:**

Autism support needs can be viewed as ‘wicked problems’, underpinned by various disrupting factors distributed across time and space. This complexity strains care providers’ ability to act swiftly and decisively. An integrative autopoietic-enactive approach may offer guidance by taking the individual’s interactional social-ecological system as the central unit of analysis. In clinical practice and research, however, this approach is only beginning to take shape. A lack of definition and codification hinders practical guidance and clinical innovation.

**Method:**

To explore what autopoietic-enactive autism healthcare might mean in practice, this scoping review maps healthcare practices that are already based on autopoietic enactivism or bear affinity with it.

**Results:**

Forty-nine publications met with inclusion, representing 2.21% of the initial corpus. Findings were synthesized using four analytical lenses: aspects of (I) *ecological-,* (II) *tangible-,* (III), *intersubjective-*, and (IV) *socio-cultural life*, representing distinguishable albeit not separable dimensions of a person’s social-ecological system.

**Conclusion:**

Our findings present a rich but still loosely integrated picture of healthcare practices. This scoping review provides a reference point from which the approach may be further defined and consolidated into a clinical research field.

## Plain Language Summary

**Introduction:**

Supporting autistic people can be challenging because their needs are complex and evolve over time. This complexity makes it difficult for care providers to determine exactly what to do. One promising way forward is autopoietic enactivism, which looks at a person in relation to their environment. In clinical practice and research, however, an autopoietic-enactive approach is still new and not clearly defined.

**Method:**

To explore what autopoietic enactivism can bring to the clinical domain, we conducted a scoping review of autism healthcare practices that already use ideas from autopoietic enactivism.

**Results:**

Out of the full body of literature that we screened, 49 publications met our criteria. These findings were brought together using four different lenses that reflect different but connected parts of everyday life: aspects of (I) *ecological-,* (II) *tangible-,* (III), *intersubjective-*, and (IV) *socio-cultural life*– or, more precisely: the everyday life domains that people live in, the material things they interact with, the other people they interact with, and the wider social and cultural context that influences these interactions.

**Conclusion:**

Our findings provide a comprehensive overview of healthcare practices, but the overall picture is not yet fully coherent. By providing an overview of existing work, this scoping review can help turn autopoietic-enactive autism healthcare into a clearer, coherent, and more well-defined research field.

## 1. Introduction

### 1.1. Autism Healthcare: *Layers of Complexity*

Autism support needs may be viewed as ‘wicked problems’,^1,2^ involving multiple layers of healthcare complexity. Well-researched is the *physiological complexity*, regarding substantial biological variation across the autism spectrum and a high rate of comorbidity.^3-5^ There is also a *psychological complexity*,^6,7^ referring to divergent variations in emotions, personality traits, and coping mechanisms, among others. De Haan adds an *existential complexity*: how reflexive self-relation shapes the course of a support need through an ‘extra loop’, such as how shame can lead to avoidance of help.^8,9^ Finally, a fourth layer is the *environmental complexity*, insofar as the outside world also plays an enabling if not constitutive role in the formation of habitual and specific goal-directed behavior. Crucially, support needs are shaped by the *continuous interplay* between these layers of complexity, across personal contexts that include educational,^10,11^ work,^12,13^ and home settings.^14,15^

Formulated differently, support needs involve various disrupting factors distributed across time and space. The causal organization between these factors is often unclear, which makes it difficult to prioritize among competing interventions^9^ - in principle, an intervention may start with any of the abovementioned layers of complexity. This complexity may undermine care providers’ capacity for timely and decisive action by amplifying uncertainty and coordination burdens. Care providers might find it difficult to determine where their responsibilities end and those of others begin, and how and when to create bridges with (in)formal support partners.^16^ At the same time, therapeutic efforts may be thwarted by breakdowns in neuromixed communication - that is: communication between people across the spectrum of neurodiversity in this case between autistic and non-autistic people -, which is as much a responsibility of the client as of the care provider.^17^

### 1.2. Autopoietic Enactivism: *An Integrative Approach*

Required here is an *integrative* account that explains not only how various factors *contribute* to a support need but also how they *cohere*.^9^ This account may be provided by *autopoietic enactivism*, a position in 4E cognition science that supports the idea that cognition *emerges* through situated and selective interaction in and with the world^18-24^ - drawing on several philosophical traditions such as pragmatism, phenomenology, ecological psychology, biological and complex systems theory.^21,25,26^ (Autopoietic enactivism represents one strand in enactivism; the other two being sensorimotor enactivism and radical enactivism.^26^ Autopoietic enactivism, the foundational strand, highlights the role of biodynamics in meaning-making; sensorimotor enactivism puts the focus on skillful environmental exploration; radical enactivism, finally, advances a fully non-representational view of cognition – seeking to ‘purge’ the former two strands from any remaining “representationalist undertones”.^26^ Despite varying emphases and philosophical disagreements, they are all “united by a common commitment to understanding cognition as rooted in our engaged, bodily lives”^26^) Two themes have been proposed as entry points for the clinical context^27^: *self-regulation* and *sense-making*.

Self-regulation refers to an individual’s ability to move within and change things in their environment in ways that support their *autonomy*. Like all organisms,^28^ human beings seek out interactions that help them thrive and avoid those that do not. For example, one might avoid an overwhelmingly busy street and find comfort under a weighted blanket. This is a *continuous* process. It is also a *subjective* process: what may be meaningful to one person is not necessarily meaningful to another, such as the weighted blanket that some may experience as helpful, yet others might find it too heavy. Each person – and each organism, for that matter - has their own *lifeworld*.

Self-regulation implies sense-making, which refers to the evaluative process that emerges from the ongoing organism-environment interaction by which aspects of the world become salient to the organism, i.e., become *meaningful* by being perceived as health-sustaining, health-diminishing, or neutral.^9,29^ This evaluative process is an *affective* activity,^9,25,29^ since health-sustaining elements are typically felt as pleasant and health-diminishing elements as unpleasant. For example, the weighted blanket is perceived as pleasant because it lowers anxiety, whereas the busy street is perceived as unpleasant because it poses danger. Combined, self-regulation and sense-making underly an organism’s ability to face an ever-changing and at times inhospitable world, towards safety and away from ill-being.

From the perspective of autopoietic enactivism, autism and other forms of neurodivergence can be viewed as *structurally atypical forms of sense-making*.^9,30^ On average, an autistic person may be more easily overwhelmed by the busy street than a non-autistic person, as they would, on average, also derive greater pleasure from a weighted blanket. Autistic *embodiment* plays a crucial role here, referring to the “particular ways in which the biology, neurophysiology, affective, and sensorimotor structures and skills of people with autism differ from those of non-autistics”.^31^ Key is that autism, as a structurally atypical form of sense-making, also introduces *atypical forms of self-regulation*. Fixed routines, for example, can help autistic individuals avoid unexpected and intense sensory events en route.^32,33^ Eye contact avoidance can be seen as a strategy to make social interactions more manageable.^31,34^ Fixed routines and eye contact avoidance, like other self-regulation strategies, can support autistic individuals in harmless and authentic ways but can also, in certain contexts, translate to difficulties and challenges in daily life. (Terminology preferences regarding person-first language - person with autism - and identity-first language - autistic person - correlate with various factors, including the strength of identification with an autism identity, age, IQ, region, and cultural background.^35-37^ For this paper, we chose to adopt identity-first language).

### 1.3. Scoping Review: *Toward Clinical Consolidation*

The potential of autopoietic enactivism is increasingly being recognized in healthcare.^9,30,38-42^ and within autism studies.^31,43-48^ It provides an integrative account that is more faithful to the complexity of autism healthcare, treating difficulties with executive functioning, social interactions, and sensory regulation not as stand-alone phenomena,^49^ but as interdependent expressions of a person’s challenges in self-regulation and sense-making. Importantly, as such, an autopoietic-enactive approach may also help navigate the ‘wickedness’ of support needs, focusing not on several competing causal explanations but by taking the person’s *interactional social-ecological system* as the central unit of analysis. The goal is to modify the human-world dynamics so that stable, authentic, and health-promoting *patterns of engagement* get established, both within and beyond healthcare settings.

In the clinical domain, however, there is no clear picture of what *autopoietic-enactive healthcare practices* may look like. Thus far, autopoietic enactivism has primarily been taken up by philosophers of psychiatry to reconceptualize psychopathology and neurodivergence through the lenses of embodiment, context, and interaction. As of yet, there is no clear overview of how these reconceptualizations can inform effective interventions in mental healthcare practice. This lack of *clinical consolidation* hinders **(1)** practical guidance for care providers to integrate various layers of complexity; **(2)**hands-on research and development; and, more broadly, **(3)** assessing and evidencing the clinical relevance of an autopoietic-enactive approach.

The present scoping review addresses this gap by surveying the literature for autism healthcare practices that connect with autopoietic enactivism - which, to our knowledge, is the first attempt to do so. We adopt a *dual focus*, identifying practices that are already based on autopoietic enactivism (*explicit)* and bear affinity with it (*implicit*). ‘Based on’ here means that the study either uses autopoietic enactivism as its main or one of its conceptual starting point(s) or draws on it to interpret the findings. It is important to consider that enactive elements may already be visible in existing healthcare practices without being recognized as such, for example in pragmatic and holistic approaches.^9^ In general, autopoietic enactivism resists straightforward definition; it is “much rather a synthesis of some new but also several old themes that mutually support each other”.^18^ Paradoxically, this scoping review aims to articulate what autopoietic-enactive healthcare practices might mean in practice, yet the very lack of clear conceptual boundaries also poses its main methodological challenge. By adopting a dual focus, we grant ourselves the necessary freedom to examine practices that are autopoietic-enactive in substance, even when not in terminology.

## 2. Method

For guidance and structure, we followed the five main stages of the scoping review framework as described by Arksey and O’Malley^50^ and Levac et al.^51^: (1) identifying the research question; (2) identifying relevant studies; (3) study selection; (4) charting the data; (5) and collating, summarizing, and reporting the results. A sixth stage is presented as well, albeit optional: consultation. This stage was not undertaken in this scoping review. Additionally, we consulted the guidelines of the Preferred Reporting Items for Systematic Reviews and Meta-Analyses extension for Scoping Review^52^– *PRISMA-ScR*.

### 2.1. Eligibility Criteria

Eligibility criteria were determined using the PCC-mnemonic^53^: *population, concept,* and *context*. To be included in the review, healthcare practices had to (1) address an autistic audience, although not exclusively. Demographic differences and the presence of comorbidity were not considered relevant. Conceptually, the focus was on (2) autopoietic enactivism, although, as mentioned, we accepted a ‘conceptual margin’ here. Contextually, the focus was on (3) formal healthcare settings, although this was not restricted to a specific domain – e.g., inpatient, ambulatory, family-based, or community-based care, etcetera.

The search scope was restricted to academic journal articles and book chapters, both peer and non-peer-reviewed, written in English. No restrictions were set regarding publication dates and study design.

### 2.2. Search Strategy and Strings

The search strategy was developed in collaboration with two information specialists. *Three searches* were conducted. The initial search took place in March 2023, conducted first with PsychINFO and later with Medline, Cinahl, Embase, Cochrane, and Web of Science. Search strings were adjusted where needed to match the commonly used terminology of each database. The second search was conducted shortly after and left out the third component – context: autism healthcare – to gauge whether the initial search was too restricted and relevant studies were missed. The third search took place in March 2025, to identify relevant publications that had been added during the intermittent period.

Search strings were organized around the eligibility criteria: “autism” AND “enactivism” AND “autism healthcare”. For autism and autism healthcare, alternative denominations included “autism spectrum disorder”, “ASD”, “mental health program evaluation”, and “therapeutic alliance”, respectively. For “enactivism”, search strings were determined iteratively in a multidisciplinary team of researchers – the authors of this paper, comprising academic and professional backgrounds in autism healthcare, philosophy of psychiatry, and linguistics. As mentioned in Section 1.3., we applied a dual focus, examining practices that are already based on autopoietic enactivism (*explicit*) and bear affinity with it (*implicit*). This is reflected in the search terms, which move beyond solely “enactiv*” but also include adjacent constructs such as related to embodied and embedded interaction, sensorimotor coordination, and intersubjective attunement.

The complete list of search terms used for PsychINFO is included in Supplementary File 1. The PRISMA-ScR checklist is included in Supplementary File 2^52^. Due to the iterative handling of search terms for the “enactivism” category, it was decided not to preregister the protocol.

### 2.3. Literature Selection

The first search resulted in 1,406 publications, of which 455 duplicates were removed. An additional 43 records were excluded for eligibility reasons. Literature selection proceeded in two phases: (1) title and abstract screening, and (2) full-text screening if necessary. This was the case for instances where compliance with the eligibility criteria was not evident and closer inspection was needed. At the title and abstract level, 908 records were screened in Rayyan (rayyan.ai), of which 861 (94.8%) were excluded for being off-topic. Forty-seven publications were sought for retrieval, of which 2 were no longer available. Full-text screening was then performed on 45 records, from which 25 were excluded for having no or too implicit engagement with autism (N = 2), autopoietic enactivism (N = 10), autism healthcare (N = 7), or the latter two combined (N = 6). Twenty publications were selected in the end, representing 1.42% of the original 1,406 records.

**Figure 1.**
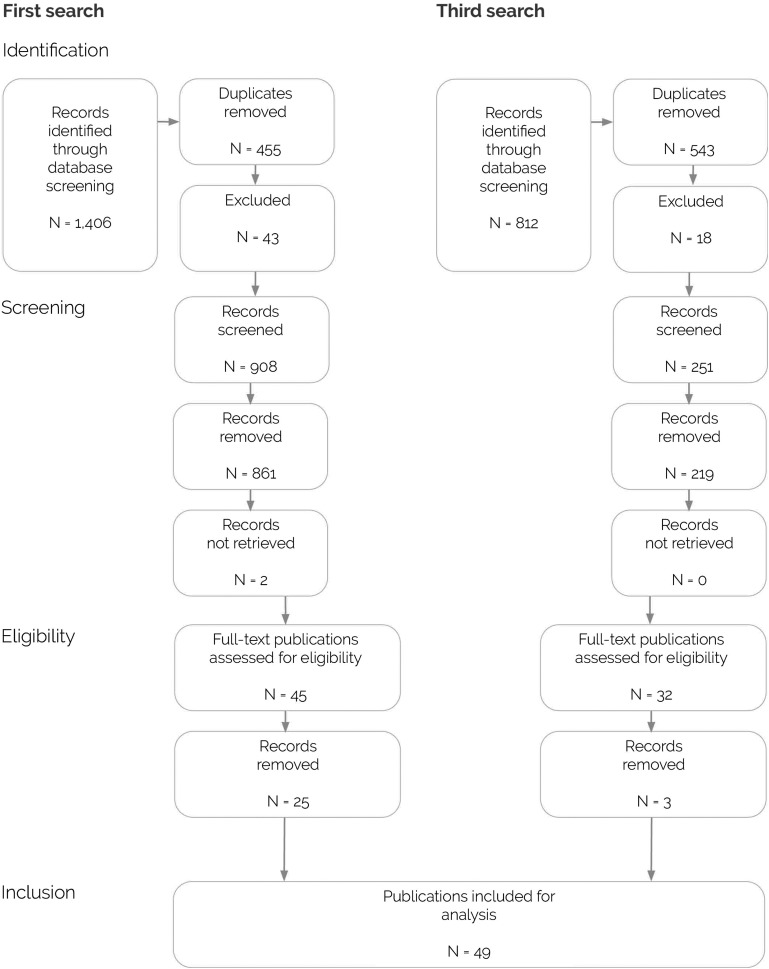
PRISMA-ScR flow diagram

The second search did not result in new publications, although the third search generated 812 records not yet identified in the first search. Following a similar procedure, 543 duplicates were removed and 18 records were excluded for eligibility reasons. Two hundred and fifty-one publications were screened at the title and abstract level, of which 219 (87.3%) records were excluded. Full-text screening was performed on all remaining 32 papers, from which 3 were excluded for having no or too little engagement with enactivism (N = 1) or autism healthcare (N = 2). Twenty-nine papers were selected in the end, representing 3.57% of the original 812 records. Combining all three searches, a total of **49 publications** (2.21%) were included in the scoping review. Figure 1 provides the PRISMA-ScR flow diagram for the first and latter search. The second search, which did not produce new results, is omitted from the figure.

All authors were involved in screening the potential publications at the title and abstract level. Full-text screening was performed by the first and third author. Doubts about inclusion were discussed among the authors and settled through consensus.

### 2.4. Charting and Synthesis

For charting, *environmental complexity* was privileged as the analytical entry point into the literature. Consequently, this scoping review focuses primarily on elements of the physical and socio-cultural environment in relation to the development and addressing of support needs, while placing less emphasis on their physiological, psychological, and existential underpinnings (Section 1.1.). This decision reflects a commitment to steer away from individualist notions of healthcare, noting that these latter layers of complexity have historically tended toward more cognitivist accounts of mental (dis)function. At the same time, from an autopoietic-enactive perspective, all four layers of complexity are understood as integrative and mutually constitutive to a person’s social-ecological system. This is also reflected in the Findings section (Section 3), which encompasses a wide range of clinical approaches – no layer of complexity has been eschewed *a priori*.

**Figure 2.**
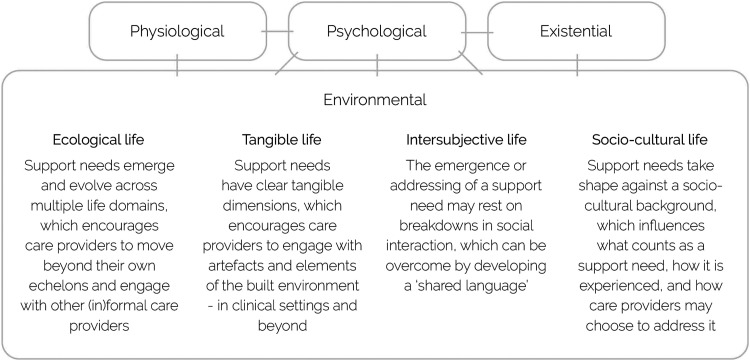
Relevant information was obtained using four lenses: aspects of ecological-, tangible-, intersubjective-, and socio-cultural life

Within the layer of environmental complexity, we further distinguished four analytical lenses: aspects of **(I)** *ecological-*, **(II)** *tangible-,* **(III)** *intersubjective-*, and **(IV)** *socio-cultural life* (Figure 2), as defined in earlier conceptual research (*under submission,*at the time of writing, a fifth lens has been added : aspects of historical life, engaging with past life events and episodes that have shaped interaction patterns in the present). These lenses are not mutually exclusive; as befits an integrative approach, any lens can be said to imply aspects of the others. It is important to consider, therefore, that we arrived at them pragmatically and iteratively: rather than prioritizing theoretical coherence, these lenses serve to conceptualize the autopoietic-enactive considerations that a care provider may encounter in day-to-day clinical practice. The challenge here, which also applies to the aforementioned layers of complexity, is to break down the autopoietic-enactive approach into conceptually and analytically manageable parts without losing sight of its fundamentally integrative nature.

Data captured through the four lenses are organized into Table 1 (Supplementary File 3). For synthesis, findings are grouped and are discussed along the same four lenses. In the text, publications that are already based on autopoietic enactivism (*explicit*) are indicated using an asterisk (*).

## 3. Findings

The literature search yielded a wide variety of study types, including clinical studies, conceptual explorations, and phenomenological analyses, encompassing both quantitative and qualitative approaches. Of these studies, 14 are already based on enactivism (*explicit*), whereas 35 bear affinity with it (*implicit*).

Study settings include (pre-)schools, therapy centers, hospitals, home contexts, and others. Geographical locations, including in scoping reviews, represent Brazil, China, South Africa, and other non-North Atlantic countries (8), although most studies are centered in Europe (31) and North America (19). Age groups range from toddlers to seniors, although the vast majority of studies focus on children and adolescents from 18 months up to and including 18 years (26), with only a few studies focusing on adults only (3) or mixed age groups (3). Overall, studies are male-skewed. Aggregating large sample-studies reporting sex/gender (N ≥ 18, totaling 647 participants^54-60^) yields a distribution of 57,5% male, 35,9% female, 5,4% non-binary/non-conforming, 0,8% other, and 0,5% missing. Notably, only Mazurek et al.^58^ provide additional detail on non-binary/non-conforming and undefined gender identities and distinguish between cis- and transgender individuals. Also in single and small-sample studies, male participants predominated. Ethnicity/race was reported almost exclusively in studies from the United States, where White/Caucasian participants predominated.

Across the corpus, sample size, sex/gender, and age distributions are not always reported consistently. A substantial portion of the corpus does not formally engage with a participant pool at all. Fourteen papers are conceptual alongside one single methodological paper,^61^ and one therapeutic handbook.^62^

### 3.1. Aspects of *Ecological Life*

***Healthcare in everyday ecologies: school*.** Several studies shift the focus from the clinical context to everyday embodied engagements - to enhance (immediate) impact and minimize the need for clinical-to-real-world skill transfer, focusing on school and home contexts.

At school, Benson et al.^63^ studied simple *in-class sensorimotor strategies* (e.g., clapping to the days of the week or passing a vibrating toy), reporting moderate improvements in pupils’ attention span and in-seat behavior. Freyone^64^ challenges the assumption that therapy must take place in a designated space, demonstrating in a drama therapy case that engaging the client in their classroom can foster safety and trust -*‘meet a client where they are’*. Everaert et al.^65^ review strategies for *embodied learning* (e.g., Dance Movement Therapy, role-play, physical activities such as running and swimming), advising integration in educational settings. Stallmann et al.^66^’s research reverses the direction, seeking to import the real-world into the therapy space. They present a *VR school scenario* that imitates peer exclusion and teacher scolding, using this to examine whether negative social experiences may be better regulated through peer support. Like Martin et al,^61^ the authors stress that VR elicits authentic situated reactions while retaining control and standardization.

***Healthcare in everyday ecologies: home*.** In the home context, Delehanty et al.^54^ analyze parent-child communication during ordinary home routines (e.g., book sharing, play, chore time), to identify where speech-language pathologists might best jump in to support *parent-implemented early interventions*. Shaughnessy et al.^59^ examined *musical supports* (e.g., handheld instruments, an echo microphone) to scaffold communication and everyday routine activities (e.g., using a song to teach tooth brushing). In the Scandinavian context, Mattsson et al.^67^ extend mentalization-based family therapy to *outdoor activities* (e.g. canoeing, hiking, art-making, nature trips), which can support attunement through shared action, and may additionally slip into daily routines.

### 3.2. Aspects of *Tangible Life*

***Spatial layout.*** Other authors zoom in on the healthcare setting itself – seeking not so much to address support needs in their real-world contexts but to understand the therapeutic exchange as an intrinsically situated process itself. Daniel et al,^62^ like other authors, emphasize the importance of *sensory stability* and the elimination of distracting stimuli. Nešić et al.^68^* invoke the medieval monastery as a *socio-material niche* whose simple sensory environment and codified rituals, routines, and social relations may have afforded autistic individuals safety and predictability. Mössler et al.^69^* examine how *room configuration* shapes opportunities for attunement. Their vignette follows an autistic boy who engages his music therapist by repeatedly throwing building blocks in the space between them. It is suggested that rearranging the room’s furniture would have made it easier for the therapist and child to ‘meet’ and connect – an interactional space whose affordances should be consciously attended to.

***Mediated interaction.*** Several studies focus not on the spatial environment but on how engagement is mediated by a third element. Mössler et al.^69^*’s abovementioned building blocks, for example, are described as opening a *channel for interaction* and coordination between the child and the music therapist. Firth et al.^70^ suggest using *familiar toys* to support Intensive Interaction sessions. Alessi^71^* discusses portrait therapy, arguing that the sheet functions as an *objectual delegate*: by drawing the other person’s face, the exchange remains affective yet dosed and under control – direct eye contact may be avoided and new possibilities for interaction are enacted. In their study on *personalized supportive technology*, Van Huizen et al.^72^* demonstrate how mediated (social) interaction can also be designed for in dedicated artefact design processes.

In three studies, the therapeutic exchange is ‘joined’ by an animal. Maresca et al.^73^ and Malcolm et al.^74^ show how *horse-assisted therapy* can elicit (surprising) displays of sociality, supported in part by the horse’s gait that provides predictable sensory stimulation. Solomon^75^ argues that a *therapy animal* changes the ‘interactional substrate’,^76^ enabling expressions of sociality and morality that are seldom visible in human-only encounters.

***Glimpsing inner worlds.*** Artefacts can also have hermeneutic value. Hart^77^ describes a client modelling an alien using Play-Doh, possibly a *metaphor* for the client’s loneliness and a potential springboard for discussion. Similarly, addressing *metaphoric engagement*, Rucińska et al.^78^* provide a vignette showing how, during a systemic therapeutic exchange between a therapist and an autistic boy, three cubes are placed as a ‘wall’ between two figurines – facilitating a dialogue about the dynamics that arise when there is conflict between him and his mother. Ferreira & Muniz^79^* examine how an *Idea Diary*– a tangible collection of photographs, captions, and drawings used in a small-group classroom morning circle – enabled autistic children to externalize their experiences, emotions, and memories, while inviting peers to engage. Finally, Bertilsson et al.^80^* focus on the *lived body* as an interpretive medium itself, arguing that the ‘movement quality’ of autistic individuals – characterized as restrained, fragmented, and hesitant – indicates emotional states and long-term bodily strategies that could inform physiotherapeutic interventions.

### 3.3. Aspects of *Intersubjective Life*

***Bodily and motor synchrony*.** A substantial number of papers focus on face-to-face engagement in neuromixed dyads through bodily tuning rather than verbal communication. Like in other studies, Fattal et al.^81^ identify interpersonal synchronization difficulties as a core challenge, and suggest *body-oriented psychotherapies* (dance-, music-, imitation-, and gesture-related therapies) as ways to improve synchrony and consequently social outcomes*. Dance Movement Therapy* (DMT) is central to four studies.^56,82-84^ DMT employs dance and movement to foster non-verbal coordination and connection, supporting emotional, cognitive, physical, and social integration without the need for spoken language. Hildebrandt et al.^56^ describe DMT-related *mirroring exercises* (Chase-Circle, dyadic mirroring, Baum-Circle), formalized in Koch & Kercher^82^’s *Mirroring Intervention Protocol*. In their *Shared Movement Approach*, Samaritter^83^* identifies joint movement as one of several *relational modes* that can arise in a DMT intervention, which the therapist may reinforce or creatively respond to.

***Rhythm and musicality*.** Bodily and motor synchrony unfold in time, and rhythm provides a structural basis for shared temporal engagement. Delafield-Butt et al.^85^ demonstrate that *simple sensorimotor exchanges* (e.g., rubs, slaps, gentle pushes) can generate a primary sense of intersubjectivity that may crescendo into moments of trust and shared joy – even in severe autism. Similarly, Hart^77^ describes how mirroring their client’s posture, gross movements, humming, clapping, and foot-tapping helped establish a shared sense of being - a form of *‘musical resonance’*. Fram et al.^55^ suggest that *parent-mediated musical approaches* can provide children with the right rhythm to practice with expressive communication. In support, Ding et al.^86^’s meta-analysis of several *rhythm-based interventions* (including music, dance, and piano) found moderate improvements in social skills and substantial gains in social interaction and emotional skills.

Rhythm furthermore has the special ability to create temporal expectations in its participants, which is strategically harnessed in Daniel^87^’s therapeutic principles to draw children into social play. During a play session, the adult matches the pulse and vitality of the child’s movements and sounds, then layers new beats that invite reciprocity. For example, the so-called *jazz gap* means to deliberately insert a slightly delayed beat to heighten anticipation, which can elicit laughter and other affective responses. Daniel et al.^62^’s *Rhythmic Relating* handbook operationalizes these principles into step-wise skill levels.

What these and the aforementioned studies aim to accomplish is what Bizzari^88^* calls an *expressive common environment*, in which the ‘intercorporeal dyssynchrony’ between care provider and client is bridged by tuning movements and lived experiences of time and space.

***Metrics of social attunement.*** Several studies harness the spatial and temporal palpability of interpersonal engagement to measure it. Samaritter & Payne^84^*’s *Social Engagement and Attunement Movement (SEAM)* helps score attunement in DMT (e.g., rhythmic synchronization, body weight usage, facial orientation). Koch & Kercher^82^ apply the *Embodied Intersubjectivity Scale*^89^ to measure how sensorimotor mimicry supports empathy. Boorom & Liu^90^’s scoping review on minimally-verbal autism maps *metrics* to measure interaction dynamics (e.g., engagement duration, counts of turn-taking).

Measuring interpersonal engagement may also be used to support the diagnostic process. Martin et al.^61^ propose an *immersive environment* that tracks sensorimotor behavior, facial expressions, and sounds, to develop a diagnostic procedure to reduce contextual and rater biases as persistently observed in human assessments. Koehler et al.^57^ trained a *machine learning diagnostic classification model* that, with various degrees of accuracy, was able to tell if there was an autistic person present in the dyad.

***Self-other differentiation and selfhood.*** Through rhythmic, bodily engagement, a sense of self is forged: it is *through* interaction with others that human beings learn how to distinguish between ‘me’ and ‘other’. Focusing on self-other differentiation through imitation, Xavier et al.^91^ recommend *peer-mediated interventions*, in which autistic children interact with communicatively proficient peers that share morphological, behavioral, and motor features. Vaisvaser^92^* argues that *Creative Arts Therapies* (CATs) – music, dance, art, drama - may help affirm and connect the nested dimensions of selfhood by offering a playful space in which aesthetic resources can help foster basic interoceptive awareness as well as explore complex emotions, memories, experiences, and self-beliefs. Although Taels et al.^60^’s phenomenological study on hypersensitivity does not address selfhood directly, it echoes the idea that *stimulating practice and activity* in therapeutic contexts can help remediate a ‘fragile sense of embodiment’ and, in turn, amplify social capabilities and connectedness.

***Verbal communication*.** Several studies - all with autistic children - address verbal breakdowns while retaining an embodied perspective, offering recommendations for sustained engagement and joint attention. Geils & Knoetze^93^ suggest matching volume and prosody, using simple and short utterances, and allowing longer pauses. Ma et al.^94^ stress that non-verbal visual, auditory, and tactile cues – *embodied resources* - may support the verbal exchange without having to repeat verbal demands. For content, Fasulo & Fiore^95^ suggest building on the child’s self-chosen topics (*tellability*), and matching the level of detail the child prefers when discussing these topics *(granularity*).

### 3.4. Aspects of *Socio-Cultural Life*

***Institutional normativity.*** Several studies attend critically to how institutional norms can steer therapeutic efforts into corrective directions, preventing autistic children to express themselves in authentic, self-chosen ways. Fasulo & Fiore^95^ show how therapeutic adult-child conversations are often structured by *didactic frameworks* that overlook the child’s meaningful contributions. Mössler et al.^69^*’s previously introduced vignette depicts a music therapist torn between attuning to a child’s musicking behavior and maintaining institutional expectations (e.g., tidiness of the room), which the authors link to a *medical professional upbringing* that is oriented toward correction rather than meaningful mutual engagement.

***Individual-level impairment*.** Particularly targeted is the biomedical tendency to locate ‘deficits’ within the individual. Turowetz and Maynard^96^ demonstrate how, for the sake of objectivity and standardization, standard diagnostic testing protocols such as the ADOS lead clinicians to obscure their own role and that of the material environment in eliciting the child’s behavior. Hajdúk et al.^97^ argue that paranoia is also too often viewed as the result of an individual-level impairment in social threat perception. Paranoia unfolds in dynamic social interactions and can be aggravated when the bodily synchrony between interaction partners falters. This may happen in neuromixed settings. The authors suggest studying a person’s paranoia in real-time and real-space, - e.g., through *geofencing* -, and examining whether *psychedelics* and *synchronization training* can help mitigate it. Jurgens^98^*, drawing on Chapman’s work on ecological functional models,^99^ adds that, while autistic traits may seem maladaptive at the individual level, they can be extra-ordinarily valuable at a collective or wider socio-ecological system level – *‘niche contribution’*, as exemplified by autistic contributions to computer culture. The author advocates a lens that enables psychotherapists to map support needs across the entire ‘organism-environment-ecology context’, which, depending on the situation, advocates environmental accommodation as much as individual adaptability.

***‘Letting be’.*** Like other authors,^72,77,100-102^ Jurgens^98^* mentions *neurodiversity* to invoke the idea that autism should be seen first and foremost as a neutral form of biodiversity – challenging wider socio-cultural standards related to conceptions of normality, competence, and human flourishing. Rather than pressurizing autistic individuals to behave according to neuro-average conventions, these studies argue that autistic ways of being and doing are legitimate and meaningful. De Jaegher^100^* condenses this stance into the notion of *letting be*, which refers to the commitment that interaction partners – autistic and non-autistic alike – remain fully themselves and embrace each other’s differences. In this regard, Mazurek et al.^58^ point out that it is crucial that therapists establish a *safe, accepting, and ‘autism-affirming’ relationship*.

***Epistemic humility and plurality.*** ‘Letting be’ is also an epistemological commitment. De Jaegher^100^* warns that distorted knowledge about autism can cause misunderstanding and harm and advocates the use of participatory practices and overarching *indigenous epistemologies*: rather than ‘fishbowling’ the autistic community in pre-informed, prejudiced ways,^103^ one may best know the other through active, interested engagement. In this regard, Murray et al.^102^* propose *phenomenological bracketing*: by purposefully suspending beliefs about each other and autism in general, neuromixed interaction partners can share and explore each other’s experiences in open dialogue.

Implied here, too, is a willingness to work with types of knowledge that traditionally lie outside the biomedical narrative. Similar to Hart,^77^ Emanuel^101^ proposes *radical empathy*, referring to autism as ‘an experience one lives’ that clinicians should approach with an open mind. The author exemplifies how they enter their client’s symbolic Pokémon universe to better understand their fears and feelings. Far from ‘fishbowling’, Vulcan^104^ describes *implicit bodily relational knowing*, where the practitioner uses their own body as an instrument for grasping an autistic child’s inner state. Park^105^ argues that clinicians need to supplement their deficit-centered biomedical lens with a *literary-philosophical grammar*, pointing out that they might otherwise overlook the meanings embedded in a child’s actions. Park further illustrates this by showing how, during a sensory integration therapy session, a boy suddenly galumphs through the room like a Tyrannosaurus rex, which they argue is as much an enactment of power and agency as a matter of proprioception. Park^106^ refers to these enactments as *embodied metaphors for intersubjectivity*.

***Power, participation, and epistemic authority.*** Intertwined here is also a shift in power dynamics: allowing the other to *become known* in authentic ways. Several studies attribute a role to the practitioner in making a sincere effort to find meaning in their client’s expressive acts, as suggested in, for example, the aforementioned studies by Geils & Knoetze,^93^ Mössler et al.^69^*, and Park.^105^ Ma et al ^94^ criticize what they call the *‘ignore’ approach'*, where attention is withdrawn to actions and utterances that seem to lack communicative intent. By contrast, Daniel^87^’s study on social play grants children the freedom to thread meaning and express themselves in *self-chosen* ways, while Ferreira & Muniz^79^*’s Idea Diary helps autistic children retain *epistemic authority*. Discussed here is the legitimacy given to stakeholder input, and, more specifically, the role and responsibility of the care provider in acknowledging this legitimacy. De Jaegher^100^* promotes the use of *participatory practices*, which consolidates the idea that autistic individuals should have a seat at the table in efforts to improve clinical practice.

## 4. Discussion

### 4.1. Summary

As mentioned at the start of this paper, support needs may be viewed as ‘wicked problems’, involving a non-linear interplay of physiological, psychological, existential, and environmental factors. This places strain on the ability of care providers to act swiftly and decisively. An autopoietic-enactive approach can help integrate these factors around the practically applicable constructs of self-regulation and sense-making, focusing on stable, authentic, and health-promoting patterns of engagement in a person’s interactional social-ecological system. Although articulate in (philosophical) theory, it remains, however, underdeveloped and poorly operationalized in the clinical domain. The present scoping review addresses this gap by surveying the literature for autism healthcare practices that connect with autopoietic enactivism - which, to our knowledge, is the first attempt to do so. In total, we studied 49 publications that are already based on autopoietic enactivism (*explicit*), or bear affinity with it (*implicit*).

In brief, for ***ecological*** ***life*** (Section 3.1.), we find an effort to embed healthcare in the everyday settings in which clients’ embodied activities and routines are enacted and shaped – primarily at school (*healthcare in everyday ecologies: school*) and at home (*healthcare in everyday ecologies: home*).

For ***tangible life*** (Section 3.2.), we find that these embodied practices – including in clinical settings – can be supported physically by (re)configuring furniture or setting sensory parameters (*spatial layout*). Artefacts, animals, and dedicated technologies can furthermore support social engagement, turning challenging human-to-human interactions into more agile triadic ones (*mediated interaction*), while artefacts such as Play-Doh, figurines, photographs, and drawings afford self-expression and shared sense-making through metaphors (*glimpsing inner worlds*).

For ***intersubjective life*** (Section 3.3.), we find that social interactions need not rely only on verbal exchange, even in human-only settings. Intersubjectivity and empathy may also be achieved through non-verbal bodily engagement, enacted through dance, movement, and mirroring (*bodily and motor synchrony*), tuning rhythms and lived temporalities (*rhythm and musicality*). These findings connect to the foundational work of Fuchs on embodied intersubjectivity and how it has been applied to research into dance and movement therapy.^43,107-109^ Social interaction here involves observable behavior that can be monitored, also for diagnostic purposes (*metrics of social attunement*). Embodied forms of social interaction may also help sharpen the boundary between ‘self’ and ‘other’, which reciprocally contributes to social life (*self-other differentiation and selfhood*). Verbal communication, when applied, may be supported by non-verbal embodied resources (*verbal communication*).

For ***socio-cultural life*** (Section 3.4.), we find that the client’s interactional social-ecological system is, by necessity, embedded in a broader socio-cultural background. Parra Rubio^110^ rightfully points out that shared processes of sense-making do not depend only on aligning communication styles, but also on the social and political structures that “legitimize non-autistic interpretations of autistic expressivity”. Stable, authentic, and health-promoting patterns of engagement may flourish or be thwarted by institutional and professional standards (*institutional normativity*). Particularly persistent is the tendency to locate ‘deficits’ within the individual, for example in diagnostic testing protocols (*individual-level impairment*). Several authors invoke the neurodiversity perspective, advocating an embrace of each other’s differences (*‘letting be’*), and getting to know autistic peers not by ‘fishbowling’ the community but through human and interested engagement (*epistemic humility and plurality*) – e.g., through participatory approaches in clinical research and practice (*power, participation, and epistemic authority*). These considerations reflect and are supported by recent philosophical discussions regarding the importance of incorporating socio-cultural and institutional (power) dynamics - which shape and constrain possibilities for interpersonal engagement - into an enactive understanding of the social challenges faced by autistic and other neurodivergent people.^38,98,110-113^

### 4.2. Methodological Considerations

Our findings should be interpreted in light of the following methodological considerations. First, as pointed out in the introduction, enactivism resists straightforward definition. This prompted us to adopt a dual focus, identifying practices that are already based on autopoietic enactivism (*explicit*) and bear affinity with it (*implicit*). This made it challenging to select search terms for the “enactivism” category and, during the inclusion process, determine a clear cut-off point for what should and what should not be considered an ‘autopoietic-enactive healthcare practice’. Different search terms and different substantive decisions may have yielded different findings. For transparency reasons, the complete list of search terms used for PsychINFO is included in Supplementary File 1.

Second, the selection of relevant publications may have been constrained by the selected databases (Section 2.2.), which were chosen to align with the scoping review’s objective of mapping applications of autopoietic enactivism in clinical contexts. Relevant publications addressing the autopoietic-enactive approach from a primarily theoretical perspective may not have been represented within these databases and may therefore not have been captured by our search strategy.

Third, for charting, this scoping review privileged environmental complexity as analytical entry point into the literature, relative to physiological, psychological, and existential layers of complexity. At the same time, from an autopoietic-enactive perspective, all four layers of complexity are considered mutually constitutive to a person’s interactional social-ecological system (Section 2.4.). Zooming out, it may be considered that the decision to foreground environmental complexity has led to disproportionate attention to the ‘embodied environment’ compared to the ‘embodying being’.^98,114^ On the one hand, we would disagree. Aspects of lived bodily engagement are discussed throughout - e.g., physical activities, sensory experiences, sensorimotor exchanges, joint movement, et cetera. Precisely from an integrative autopoietic-enactive perspective, embodiment is continuously implied in thinking about and describing interactions in ecological, tangible, intersubjective, and socio-cultural contexts. On the other hand, we agree that little attention has been paid to, for example, biological and physiological structures and processes. A notable omission in this regard are autopoietic-enactive interpretations of pharmacological interventions.^40^ Importantly, however, such publications were not deliberately excluded (Section 2.4); rather, they were not identified, suggesting a research gap rather than a methodological omission.

## 5. Conclusion

To conclude, autopoietic enactivism is a conceptually rich and holistic framework that does not allow for neat, clear-cut definition and operationalization. This carries over in the present review, which presents a rich but loosely integrated picture of healthcare practices. It is challenging to extract a central thread, and more practices may be considered autopoietic-enactive than currently captured by it. The scoping review presented in this paper should not be understood as a definitive account, but rather a starting point for clinical consolidation. Autopoietic-enactive autism healthcare is in its infancy, in need of definition and codification. Clinical consolidation will help ground **(1)** practical guidance for care providers to integrate various layers of complexity; **(2)**hands-on research and development; and, more broadly, **(3)** assessing and evidencing its clinical relevance. This scoping review provides an overview of existing work – a *reference point* with ample examples.

## Supplemental Material

Supplemental Material - Enactivism in Autism Healthcare Practices: A Scoping Review Supplemental Material for Enactivism in Autism Healthcare Practices: A Scoping Review by Johannes Cornelis van Huizen, Jan-Pieter Teunisse, Rosa H. van Doormaal, Antje Orgassa, Wouter G. Staal, Derek W. Strijbos in INQUIRY: The Journal of Health Care Organization, Provision, and Financing

Supplemental Material - Enactivism in Autism Healthcare Practices: A Scoping Review Supplemental Material for Enactivism in Autism Healthcare Practices: A Scoping Review by Johannes Cornelis van Huizen, Jan-Pieter Teunisse, Rosa H. van Doormaal, Antje Orgassa, Wouter G. Staal, Derek W. Strijbos in INQUIRY: The Journal of Health Care Organization, Provision, and Financing

Supplemental Material - Enactivism in Autism Healthcare Practices: A Scoping Review Supplemental Material for Enactivism in Autism Healthcare Practices: A Scoping Review by Johannes Cornelis van Huizen, Jan-Pieter Teunisse, Rosa H. van Doormaal, Antje Orgassa, Wouter G. Staal, Derek W. Strijbos in INQUIRY: The Journal of Health Care Organization, Provision, and Financing

## Data Availability

All data generated or analyzed during this study are included in this published article and its supplementary files.*
