# Supplementary material for: Enactivism in Autism Healthcare Practices: A Scoping Review
Source: Inquiry. 2026 Jul 13;63:00469580261467452. doi: 10.1177/00469580261467452 (PMC13365672; doi:10.1177/00469580261467452)
Supplement: Supplemental Material - Enactivism in Autism Healthcare Practices: A Scoping Review [file sj-pdf-1-inq-10.1177_00469580261467452.pdf]

Supplementary File 1

PsychINFO version 29-03-2023

Autism

S1 DE "Autism Spectrum Disorders" OR TI(Autis* OR (Pervasiv* N1 Develop* N1 disorder*) OR Asperger* OR ASD) OR AB(Autis* OR (Pervasiv* N1 Develop* N1 disorder*) OR Asperger* OR OR ASD) OR SU(Autis* OR (Pervasiv* N1 Develop* N1 disorder*) OR Asperger* OR ASD)

Enactivism

S2 TX(enactiv* OR enaction OR (participatory N1 sense N1 mak*) OR (shared W1 (understand* OR interact*)))

S3 DE "Interpersonal Interaction" OR TI(((Coordination OR attun*) N1 dynamic*) OR (social N1 coordinat*) OR (interpersonal N1 (interact* OR coordinat*)) OR ((interactional OR interpersonal OR behaviour* OR behavior OR affect* OR bodi* OR movement* OR social* OR temporal*) N1 synchron*) OR (intersubject* N1 (engag* OR attunement)) OR (autonomy N3 interact*)) OR AB(((Coordination OR attun*) N1 dynamic*) OR (social N1 coordinat*) OR (interpersonal N1 (interact* OR coordinat*)) OR ((interactional OR interpersonal OR behaviour* OR behavior OR affect* OR bodi* OR movement* OR social* OR temporal*) N1 synchron*) OR (intersubject* N1 (engag* OR attunement)) OR (autonomy N3 interact*)) OR SU(((Coordination OR attun*) N1 dynamic*) OR (social N1 coordinat*) OR (interpersonal N1 (interact* OR interpersonal OR coordinat*)) OR ((interactional OR behaviour* OR behavior OR affect* OR bodi* OR movement OR social* OR temporal*) N1 synchron*) OR (intersubject* N1 (engag* OR attunement)) OR (autonomy N3 interact*))

S4 TI((Mutual OR interpersonal) N1 (connectedness OR embeddedness OR coordination)) OR AB((Mutual OR interpersonal) N1 (connectedness OR embeddedness OR coordination)) OR SU((Mutual OR interpersonal) N1 (connectedness OR embeddedness OR coordination))

S5 TI(embodi* OR ((Enact* OR Extended OR embedded) N1 cognit*)) OR AB(embodi* OR ((Enact* OR Extended OR embedded) N1 cognit*)) OR SU(embodi* OR ((Enact* OR Extended OR embedded) N1 cognit*))

S6 DE "Intersubjectivity" OR DE "Intersubjectivity (Philosophy)" OR DE "Synchrony" OR

TI(intersubject* OR synchron* OR attun* OR dyadic*) OR SU(intersubject* OR synchron* OR attun* OR dyadic*)

S7 DE "Social Interaction" OR DE "Social Behavior" OR DE "Social Cognition" OR DE "Social Connectedness" OR TI(((social OR interpersonal*) N1 (interact* OR connect*)) OR intercorporealit* OR intercorporalit* OR (social N1 (behaviour* OR behavior* OR cognition))) OR AB(((social OR interpersonal*) N1 (interact* OR connect*)) OR intercorporealit* OR intercorporalit* OR (social N1 (behaviour* OR behavior* OR cognition))) OR SU(((social OR interpersonal*) N1 (interact* OR connect*)) OR intercorporealit* OR intercorporalit* OR (social N1 (behaviour* OR behavior* OR cognition)))

S8 S6 AND S7

S9 TI(((sensorimotor* OR sensorymotor* OR "sensori motor" OR "sensory motor") N1 (coordinat* OR respons*)) OR (social N1 motor* N1 coordinat*)) OR AB(((sensorimotor* OR sensorymotor* OR "sensori motor" OR "sensory motor") N1 (coordinat* OR respons*)) OR (social N1 motor* N1 coordinat*)) OR SU(((sensorimotor* OR sensorymotor* OR "sensori motor" OR "sensory motor") N1 (coordinat* OR respons*)) OR (social N1 motor* N1 coordinat*))

S10 TI((mind N3 body N3 world N3 topolog*) OR (agent* N3 environment* N3 relation*) OR (Interrelation N3 body N3 mind N3 world)) OR AB((mind N3 body N3 world N3 topolog*) OR (agent* N3 environment* N3 relation*) OR (Interrelation N3 body N3 mind N3 world)) OR SU((mind N3 body N3 world N3 topolog*) OR (agent* N3 environment* N3 relation*) OR (Interrelation N3 body N3 mind N3 world))

S11 S2 OR S3 OR S4 OR S5 OR S8 OR S9 OR S10

S12 S1 AND S11

Healthcare setting and related; therapeutic mechanisms- and alliance

S13 DE "Treatment Effectiveness Evaluation" OR DE "Mental Health Program Evaluation" OR TI(therap* OR "Treatment Effectiveness Evaluation" OR "Mental Health Program Evaluation" OR (Treatment W1 satisfaction)) OR AB(therap* OR "Treatment Effectiveness

Evaluation" OR "Mental Health Program Evaluation" OR (Treatment W1 satisfaction)) OR SU(therap* OR "Treatment Effectiveness Evaluation" OR "Mental Health Program Evaluation" OR (Treatment W1 satisfaction))

S14 DE "Therapeutic Alliance" OR TI(Alliance* OR therap* OR intervention*) OR AB(therap*) OR SU (Alliance* OR therap*) OR TI((Patient N1 (client OR therapist*) N1 relation*) OR (treat* N1 (outcome* OR result*)) OR ("Life span*" N1 (perspective OR care)) OR (Network* N1 (perspective* OR care)) OR (Transitional N1 (care OR (healthcar*) OR (health N1 care))) OR (Mental N1 (healthcare OR "health care")) OR ((Outpatient* OR "out patient*") N1 care)) OR AB((Patient N1 (client OR therapist*) N1 relation*) OR (treat* N1 (outcome* OR result*)) OR ("Life span*" N1 (perspective OR care)) OR (Network* N1 (perspective* OR care)) OR (Transitional N1 (care OR (healthcar*) OR (health N1 care))) OR (Mental N1 (healthcare OR "health care")) OR ((Outpatient* OR "out patient*") N1 care)) OR SU((Patient N1 (client OR therapist*) N1 relation*) OR (treat* N1 (outcome* OR result*)) OR ("Life span*" N1 (perspective OR care)) OR (Network* N1 (perspective* OR care)) OR (Transitional N1 (care OR (healthcar*) OR (health N1 care))) OR (Mental N1 (healthcare OR "health care")) OR ((Outpatient* OR "out patient*") N1 care))

S15 TI(mechanism* OR ((Therapeutic OR active OR common OR change OR helpful or specific) N1 factor*) OR (Change N1 process*) OR (key N1 ingredient*)) OR AB(mechanism* OR ((Therapeutic OR active OR common OR change OR helpful OR specific) N1 factor*) OR (Change N1 process*) OR (key N1 ingredient*)) OR SU(mechanism* OR ((Therapeutic OR active OR common OR change OR helpful OR specific) N1 factor*) OR (Change N1 process*) OR (key N1 ingredient*))

S16 S13 OR S14 OR S15

S17 S12 AND S16
