# Supplementary material for: Enactivism in Autism Healthcare Practices: A Scoping Review
Source: Inquiry. 2026 Jul 13;63:00469580261467452. doi: 10.1177/00469580261467452 (PMC13365672; doi:10.1177/00469580261467452)
Supplement: Supplemental Material - Enactivism in Autism Healthcare Practices: A Scoping Review [file sj-pdf-3-inq-10.1177_00469580261467452.pdf]

Supplementary File 3

Table 1. Charting table of the included publications.

Authors, publication year, location<br>(*) means explicit connection to autopoietic enactivism	**Study type and aim**	***Sample characteristics, if provided (N, age, sex/gender, ethnicity/race, (clinical) settings, additional diagnostic information)***	***Ecological life***	***Tangible life***	***Intersubjective life***	***Socio-cultural life***
Alessi (2024)*<br><br>**Location**<br>Not applicable (hereafter: NA)	A theoretical paper that examines how portrait therapy can support autistic individual's cognitive-perceptual competences by guiding how they engage with faces	***N:*** *NA*<br>***Age:*** *NA*<br>***Sex/gender:*** *NA*<br>***Ethnicity/race:*** *NA*<br>***(Clinical) setting:*** *NA*<br>***Additional diagnostic information:*** *NA*		*Objectual delegates*<br>Portraits involve 'objectual delegates' such as drawing sheets that mediate social interactions in a standardized, predictable, and manageable way	*Semiotic field of affordances*<br>Shared drawing makes social interaction manageable through predictable actions, gazes, and pauses	*Niches of norms*<br>Portrait therapy operates within structured niches of norms, where social expectations and material practices serve to create a viable and less uncertain environment
Benson et al. (2019)<br><br>**Location:**<br>USA	A quasi-experimental study to examine the effects of sensorimotor strategies on attention and in-seat behavior	***N:*** *3*<br>***Age:*** *4 - 5 (M: 4.5)*<br>***Sex/gender:*** *male*<br>***Ethnicity/race:*** *Caucasian*<br>***(Clinical) setting:***<br>*Integrated*	*Sensorimotor strategies*<br>In-class sensorimotor strategies, such as stomping feet and clapping to the days of the week, can help children modulate sensory responses	*Vibrating toys*<br>Vibrating toys as a sensorimotor strategy to enhance classroom participation		*Team-based adaptation*<br>Therapists should collaborate with educational teams to identify causes of problematic behavior and tailor interventions

		preschool classroom<br>***Additional diagnostic information:*** *Verbal*	and improve classroom participation and in-seat behavior<br>*In-class embeddedness*<br>The child receives the intervention in their natural learning environment, embedded in school activities and routines			*Low-cost and non-invasive*<br>There is a low threshold for sensorimotor strategies to be integrated into school activities
Bertilsson et al. (2022)*<br>**Location:**<br>Sweden	A descriptive phenomenological study to explore physiotherapists' lived experiences of movement quality in autism	***N:*** *10 physiotherapists*<br>***Age:*** *43 – 62*<br>***Sex/gender:*** *Female*<br>***Ethnicity/race:*** *Not provided (hereafter: NP)*<br>***(Clinical) setting:***<br>*Physiotherapy*<br>***Additional diagnostic information:*** *NA*		*'Reading' the lived body*<br>Recognizing embodied responses as restrained, fragmented, and hesitant opens opportunities for therapists to tailor interventions and foster bodily self-awareness<br>*Perform new movements*<br>Therapists can gently guide individuals to try new movements in small steps, strengthening inner 'bodily resources' – ultimately, expanding their range of movement and bodily expression		
Bizzari (2023)*<br>**Location:**	A theoretical-phenomenological explication that explores the concept of the lived body in mental pathologies	***N:*** *NA*<br>***Age:*** *NA*<br>***Sex/gender:*** *NA*<br>***Ethnicity/race:*** *NA*		*Lived time and lived space*<br>Therapists should take into account that the client can have a different experience of	*Expressive common environment*<br>Intersubjective exchanges between a therapist and autistic client should be	*Acknowledge the 'biographically determined situation'*<br>Therapists must not merely analyze the client in their social role;

NA	(schizophrenia, depression, autism) and implications for therapy	***(Clinical) setting:*** *NA*<br>***Additional diagnostic information:*** *NA*		temporality and spatiality, which can disrupt social participation	characterized by a 'shared common environment', including pre-linguistic features, such as seen in movement and music therapy	recognize them as a unique individual
Boorom & Liu (2024)<br>**Location:**<br>NP	A scoping review to identify and synthesize methods and findings from studies on interaction dynamics in minimally-verbal individuals	***N, aggregated:*** *63, across 7 studies*<br>***Age:*** *2 – 18*<br>***Sex/gender:*** *NP*<br>***Ethnicity/race:*** *NP*<br>***(Clinical) setting:*** *NA*<br>***Additional diagnostic information:***<br>*Minimally-verbal*			*Metrics for non-verbal interaction dynamics*<br>Using metrics such as turn-taking, reciprocity, and social synchrony help track how minimally-verbal autistic individuals engage and co-construct meaning	*Double empathy problem*<br>Breakdowns in interpersonal engagement should be studied in dyads, not solely at the side of the autistic individual
Daniel (2019)<br>**Location:**<br>NA	A theoretical paper that applies communicative musicality to play therapy with children, offering therapeutic principles that focus on rhythm and intentional movement	***N:*** *composite case of several children*<br>***Age:*** *children*<br>***Sex/gender:*** *NP*<br>***Ethnicity/race:*** *NP*<br>***(Clinical) setting:*** *non-directive play therapy*<br>***Additional diagnostic information:***<br>*NP*			*Rhythmic patterns in play therapy*<br>Play therapists can use rhythmic patterns, vitality-matching, and musical concepts to support social exchanges and invigorate spontaneity through social anticipation	

Daniel et al. (2024)<br><br>**Location:**<br><br>NA	A practical handbook that provides step-by-step guidelines and examples to support shared social timing through play	***N:*** *NA*<br><br>***Age:*** *NA*<br><br>***Sex/gender:*** *NA*<br><br>***Ethnicity/race:*** *NA*<br><br>***(Clinical) setting:*** *Occupational therapy, Speech Therapy, Physiotherapy, Creative Arts Therapies*<br><br>***Additional diagnostic information:***<br><br>*NA*		*Sensory stability*<br><br>Rapport can only be built in a sensory-friendly environment, which can be established through environmental adaptations such as by keeping the place tidy and odor-free	*Rhythmic Relating Handbook*<br><br>A structured approach to building interpersonal synchrony and shared timing through play. Practitioners are guided to mirror and match the child's expressions and behaviors<br><br>*'Copy the offer'*<br><br>Copying the offer can be done through different skills levels: direct mirroring (level 1), rhythm and vitality matching (level 2), and the use of activation contours and accents (level 3)	
De Jaegher (2023)*<br><br>**Location:**<br><br>NA	A theoretical paper that develops an engaged epistemology for understanding how autistic and non-autistic individuals interact across difference	***N:*** *NA*<br><br>***Age:*** *NA*<br><br>***Sex/gender:*** *NA*<br><br>***Ethnicity/race:*** *NA*<br><br>***(Clinical) setting:*** *NA*<br><br>***Additional diagnostic information:***<br><br>*NA*				*'Letting be'*<br><br>Begin from how people are and let them be who they are – this is an ontological, epistemological, and ethical commitment<br><br>*Engaging, indigenous epistemology*<br><br>Adopt an indigenous epistemology to resist colonial 'othering' and overall misunderstanding<br><br>*Participatory practices and design*<br><br>Involve autistic people

					as co-investigators in research, practice, and design, to ensure relevance, person-world fit, and explore notions of empowerment<br><br>*Narrative journeys*<br><br>Attend to autistic individual's lived experiences and personal stories
Delafield-Butt et al. (2020)<br><br>**Location:**<br><br>UK	A qualitative study drawing on a single case to examine primary intersubjective exchanges within Intensive Interaction	***N:*** *1*<br><br>***Age:*** *18*<br><br>***Sex/gender:*** *Female*<br><br>***Ethnicity/race:*** *NP*<br><br>***(Clinical) setting:*** *daily educational resource center*<br><br>***Additional diagnostic information:***<br><br>*Severe autism, non-verbal, psychologically and emotionally distant, display of stereotypies and extremely aggressive behavior*		*Motor imitation*<br><br>Join the client in their movements, sounds, and rhythms, to create trust and improve attunement, such as seen in Dance / Movement Therapy<br><br>*Primary intersubjective exchanges*<br><br>Establish meaningful communicative exchanges by using simple, expressive gestures such as rubs, slaps, etc.<br><br>*Narrative meaning-making*<br><br>Neuromixed interaction can follow a narrative arc even without language: interaction begins, intensifies, climaxes, and resolves, through rhythmic,	

					embodied engagements	
Delehanty et al. (2024)<br><br>**Location:**<br><br>USA	An observational study that examines patterns of child communication and parent verbal responsiveness across everyday interaction contexts	***N:*** *211 in total, 121 autistic, 46 with developmental delay, 44 with typical development*<br><br>***Age:*** *18 – 24 months (M: 20.3, SD: 2.0)*<br><br>***Sex/gender:*** *autistic: 86% male*<br><br>***Ethnicity/race:*** *autistic: 73% White, 18% Black, 7% more than one race; 3% Asian, 12% Hispanic*<br><br>***(Clinical) setting:*** *home environment*<br><br>***Additional diagnostic information:***<br><br>*NP*	*Parent-implemented, family-centered early intervention*<br><br>Everyday activities such as chores and eating, which are predictable and provide structure, provide built-in opportunities for parents to support their children's communication and language learning. This decreases the need to generalize social skills<br><br>*Joint attention*<br><br>Opportunities for joint attention may arise in naturalistic settings, and there is no need for caregivers to focus strongly on achieving communicative targets<br><br>*Collaboration between speech-language pathologists and caregivers*<br><br>Speech-language therapists and early intervention providers partner with families to select meaningful routines and embed strategies to support communication and		*CSBS Developmental Profile*<br><br>A method to identify child communicative acts as gestures, vocalizations, or words that were intentionally directed at another person, and with communicative intent	

			language development			
Ding et al. (2024)<br><br>**Location:**<br><br>Various: Germany (4x), USA (2x), Brazil, Canada, Iran, India, Romania	A systematic review and meta-analysis to explore the effects of rhythm-based interventions on overall social skills, social interaction, communication, and emotion	***N, aggregated:*** *408*<br><br>***Age:*** *range from preschool children to adults*<br><br>***Sex/gender:*** *NP*<br><br>***Ethnicity/race:*** *NP*<br><br>***(Clinical) setting:*** *various, including music training, dance / movement therapies, piano therapies*<br><br>***Additional diagnostic information:***<br><br>*NP*			*Rhythm-based interventions*<br><br>Rhythm-based interventions, including music, dance, drumming, and piano, support social interaction, enhance connection between autistic individuals and others, and promote social bonding and prosocial behavior	
Emanuel (2015)<br><br>**Location:**<br><br>USA	A theoretical paper drawing on two vignettes that uses contemporary psychoanalysis to frame autism as a lived experience, focusing on intersubjective relating and radical empathy	***N:*** *1 composite case (Joey), 1 individual case (Serena)*<br><br>***Age:*** *young adults*<br><br>***Sex/gender:*** *Joey: NP, Serena: female*<br><br>***Ethnicity/race:*** *NP*<br><br>***(Clinical) setting:*** *NP*<br><br>***Additional diagnostic information:***<br><br>*NP*			*Subtle interpersonal bids*<br><br>Attend to the 'subtle interpersonal bids' to support intersubjective exchanges	*Phenomenological orientation*<br><br>Understand autism as a lived experience, not only as a set of decontextualized and observable behaviors<br><br>*Radical empathy*<br><br>Enter the client's symbolic world with openness and humility, to better understand their history, emotions, and concerns<br><br>*Improvisational psychoanalytic*

						approach<br>Play and symbolic material, engaged in flexible and spontaneous ways, can help the therapeutic dyad access inner experience and foster relational development
Everaert et al. (2024)<br>**Location:**<br>Various: Europe (5x), USA (4x), Canada (2x), China, United Kingdom (listed separately from 'Europe')	A rapid review that examines the relationship between embodied cognition, well-being, and academic performances in educational contexts	***N, aggregate:*** *NP*<br>***Age:*** *school-aged individuals (pre-, primary-, secondary-, and high school)*<br>***Sex/gender:*** *NP*<br>***Ethnicity/race:*** *NP*<br>***(Clinical) setting:*** *NP*<br>***Additional diagnostic information:***<br>*NP*	*Embodied learning*<br>Physical activities, such as swimming, running, and cycling, can foster learning by improving motor competences, social skills, emotional expression, and joint attention		*Social connectivity*<br>Embodied interventions such as Dance / Movement Therapy can enhance social connectivity	*Integration in educational settings*<br>Practitioners and policymakers are advised to review how embodied learning strategies can be integrated into educational settings
Fasulo & Fiore (2007)<br>**Location:**<br>Italy	A theoretical paper drawing on two case studies demonstrating how didactic focus on correctness can hinder spontaneous talk and recognition in adult-child conversations	***N:*** *2*<br>***Age:*** *10 and 13*<br>***Sex/gender:*** *male*<br>***Ethnicity/race:*** *NP*<br>***(Clinical) setting:*** *therapy center, 'Time for Talking'*<br>***Additional diagnostic information:*** *high functioning autism, difficulties with*			*Tellability, granularity, and sequential orientation*<br>Therapists should attend to topics that inspire the child to engage (tellability), appropriate level of detail (granularity), and natural conversation flow (sequential orientation).<br>*Conversation tools*	*'Uninformed recipient'*<br>Therapist can assume the role of uninformed recipient to support the agency of children in adding to the conversation. Avoid rigid question-answer structures and baby-talk; trust children's communicative competences<br>*Avoid didactic*

		non-literal language			Mechanisms such as continuers and pauses demonstrate attention and interest	*frameworks*<br>Spontaneous dialogue should be prioritized over school-like norms and interrogative formats
Fattal et al. (2025)<br>**Location:**<br>NA	A conceptual review that examines how disrupted interpersonal synchronization may affect the development of social cognition in autism and schizophrenia	***N:*** *NA*<br>***Age:*** *NA*<br>***Sex/gender:*** *NA*<br>***Ethnicity/race:*** *NA*<br>***(Clinical) setting:*** *NA*<br>***Additional diagnostic information:***<br>*NA*	*Implementation at at-risk families*<br>Rhythm- and imitation-based interventions may be most effective when implemented early in families at risk of autism		*Body-oriented psychotherapy*<br>Umbrella term for a cluster of therapies that address dis-embodiment by enhancing awareness of bodily, emotional, and perceptual experiences<br>*Rhythm-, music-, and dance-based therapies*<br>Rhythm-, music-, and dance-based therapies can strengthen intra-individual synchronization, motor competences and executive functioning<br>*Mirroring behaviors*<br>(Parent-implemented) interventions that target mirroring behaviors may increase non-verbal social interaction, joint attention, and shared affect<br>*Gesture-based interventions*<br>Gesture-based	*Interpersonal synchrony training in (clinical) settings*<br>Training clinicians and supervisors to engage in mimicry and synchronization may enhance the therapeutic effort

					interventions can improve fine motor skills, gesture recognition and use, verbal imitation, play and social engagement, and language use	
Ferreira & Muniz (2024)**<br><br>**Location:**<br><br>Finland and Brazil	A theoretical paper drawing on two cases that shows how material engagement can enable autistic children to express ideas, interests, and experiences, supporting in-class peer engagement	***N:*** *2*<br><br>***Age:*** *10 (Alan) and 9 (Henrique)*<br><br>***Sex/gender:*** *male*<br><br>***Ethnicity/race:*** *NP*<br><br>***(Clinical) setting:*** *Regular classroom (Brazil) and special education classroom (Finland)*<br><br>***Additional diagnostic information:***<br><br>*Alan: repetitive behaviors, social interaction difficulties, sensorial sensitivity; Henrique: non-verbal, hypersensitivity to changes and overstimulating environments*		*Idea Diary*<br><br>Children can use multimodal materials, such as drawings and photographs, to express ideas and experiences	*Peer engagement and connection*<br><br>By discussing the Idea Diary in class, classmates are able to join in processes of shared sense-making	*Epistemic authority*<br><br>Children retain epistemic authority of the lived knowledge that is expressed through the Idea Diary
Firth et al. (2021)<br><br>**Location:**	A qualitative study that explores conscious and intuitive decision-making of Intensive Interaction	***N:*** *13 physiotherapists*<br><br>***Age:*** *NP*<br><br>***Sex/gender:*** *NP*	*Proactive environmental preparation*<br><br>Keep the physical set-up calm; rearrange if	*Familiar objects and language to scaffold interaction*<br><br>Objects and language are used to create	*Responsive engagement*<br><br>Practitioners begin by quietly sharing the space without pressure	*Professional reflexivity*<br><br>Embedding structured reflection and collaborative review deepens understanding

UK	practitioners when working with autistic and/or clients with intellectual disability	***Ethnicity/race:*** *NP*<br><br>***(Clinical) setting:*** *clinical, educational, and home contexts*<br><br>***Additional diagnostic information:*** *NA*	necessary. The presence of other people must be considered<br><br>*Personal history*<br><br>Consider family, residential and diagnostic factors that may shape how the client perceives and engages with Intensive Interaction	recognizable, comfortable contexts for interaction	or demands. They wait for behavior from the client that suggests openness to interaction, and then engage responsively to verbal and non-verbal signals – using well-placed pauses, and a mutually agreed tempo or rhythm. In addition, practitioners consider signs – such as gaze aversion – that suggest the client wishes to disengage	of the practice and the client<br><br>*Reflection on cultural or social issues*<br><br>Intensive Interaction can shape broader cultural and social attitudes
Fram et al. (2024)<br><br>**Location:**<br><br>USA	A quantitative study with autistic and non-autistic toddlers to examine how musical rhythm engagement relates to expressive communication, and if this can be harnessed by parent-child musical interactions	***N:*** *123 in total, 74 autistic, 49 non-autistic*<br><br>***Age:*** *14 – 36 months (autistic: M: 28.3, SD: 5.3)*<br><br>***Sex/gender:*** *60 male, 14 female*<br><br>***Ethnicity/race:*** *autistic: 60,8% Caucasian, 14,9% Black, 1.4% Asian, 23,0% Mixed race, 81,9% not Hispanic*<br><br>***(Clinical) setting:*** *home contexts*<br><br>***Additional diagnostic information:***<br><br>*NA*	*Parent-mediated music interventions*<br><br>A musical environment at home can contribute to expressive communication skills, and may be integrated with Parent-Early Start Denver Model or parent-mediated Reciprocal-Imitation Training		*Rhythm-based musical interventions*<br><br>Rhythm synchronization abilities relates to social skills, which may be fostered through musical interventions<br><br>*Assessment tools*<br><br>The Vineland Adaptive Behavior Scales, Communication and Interpersonal Relationships subdomains[1] were used to measure the child's communication and social skills; the Music@Home-Infant was used to capture the musical environment at home	
Freyone (2024)	A qualitative paper drawing on a single case study that	***N:*** *1*<br><br>***Age:*** *13*	*'Meeting a client where they are' - literally*<br><br>To foster trust and a	*Non-verbal communication supports*<br><br>Using a mirror to make	*'Meeting a client where they are' - figuratively*<br><br>Establish connection	*Child-led therapy*<br><br>Do not impose the educational setting's

**Location:**<br><br>NP	describes how embodied experiences of togetherness underpin the development of trust	***Sex/gender:*** *female*<br><br>***Ethnicity/race:*** *NP*<br><br>***(Clinical) setting:*** *in-house drama therapy service in a special educational needs and disabilities school*<br><br>***Additional diagnostic information:*** *concurrent: pathological demand avoidance, difficulties with sensory processing, physical closeness, verbal communication*	sense of safety, the therapist might have to 'relocate' to the safe space of the child; 'meet a client where they are'<br><br>*Cultivate 'newness' for the client*<br><br>Merge novel therapy activities with pre-existing activities in familiar environment, offering the client a sense of safety and security<br><br>*Multi-stakeholder coordination*<br><br>Collaboration with school staff and caregivers can help sustain therapeutic strategies and consistent application across settings	eye contact; using communication cards<br><br>*Purpose-built room*<br><br>Therapy was supported by a ritualised visual timetable in a designed space, offering a 'contained and structured space'	and/or support verbal communication in non-verbal ways, for example by mirroring body language	demands and routines, nor the therapist's agenda on the child; safeguard their autonomy
Geils & Knoetze (2008)<br><br>**Location:**<br><br>South Africa	A qualitative study that employs conversation analysis of an autistic boy in a play-based intervention to identify practices that support or hinder coordinated interaction	***N:*** *1*<br><br>***Age:*** *6*<br><br>***Sex/gender:*** *male*<br><br>***Ethnicity/race:*** *NP*<br><br>***(Clinical) setting:*** *play-based intervention program*<br><br>***Additional diagnostic information:*** *Pervasive*			*Strategies and devices for coordinated interaction, including: language use and structure in verbal communication;*<br><br>Short, simple utterances and turn completions reduce pressure and enable richer exchanges. Repetition is viewed as meaningful participation	*Use of conversation analysis*<br><br>Can reveal subtle interactional complexities that would be overlooked by approaches that treat the individual as inherently deficient; it reframes discourse into a matter of collaboration and mutual responsibility

Developmental Disorder (Autistic Spectrum)

Multi-modal and non-verbal communication;

Combining verbal cues with non-verbal cues reduces pressure to speak and leads to more spontaneous speech. Physical affection, e.g. gentle touches, can support and sustain engagement when verbal communication is difficult

Playfulness;

Playful styles of communication – gamifying, silly sounds, tickling, etc. - increases enjoyment and engagement, making the interaction more appropriate for children

Responsiveness

Matching the child's communication style, e.g. using a soft voice or whispering, can make the child feel safe and understood; it also minimizes sensory overload

Strategies and devices to avoid

The use of long, complex, or abstract utterances, rapid

					streams of (verbal) questions or demands; adopting a directive or controlling communication style that does not recognize the child's (non-verbal) contributions, nor their attempts to initiate interactions or take a turn; establishing interaction in a sensorily overwhelming space	
Hajdúk et al. (2024)<br><br>**Location:**<br><br>NA	A theoretical paper that reframes paranoia as an interactional phenomenon	***N:*** *NA*<br><br>***Age:*** *NA*<br><br>***Sex/gender:*** *NA*<br><br>***Ethnicity/race:*** *NA*<br><br>***(Clinical) setting:*** *NA*<br><br>***Additional diagnostic information:*** *NA*	*Unobtrusive tracking of social interactions, possibly using geofencing*<br><br>Track real-world interactions via geofencing – which uses GPS data to see if someone is likely to be interacting - , and 'ecological-momentary-assessments', which gather in-the-moment reports on interaction dynamics	*Administration of psychedelics such as MDMA*<br><br>If atypical synchronization patterns are contributory to paranoia, MDMA as a pharmacological intervention can lead to increased joyful experiences of interaction	*Actor-Partner Interdependence Model*[2]<br><br>Use the Actor-Partner Interdependence model as an analytical tool to examine how the paranoid person, the interaction partner, and the interaction itself influences the social dynamics<br><br>*Non-pharmacological synchronization training*<br><br>Social connection can be enhanced by bodily and motor coordination training, e.g. as seen in dance, martial arts, or imitation-based exercises	*Relational framing of paranoia*<br><br>Understand paranoia as arising in real-time interpersonal misattunement rather than caused by isolated social-cognitive deficits
Hart (2024)<br><br>**Location:**<br><br>UK	A theoretical paper drawing on a single case that explores embodied mirroring as a means to bridge perceptual	***N:*** *1*<br><br>***Age:*** *13*<br><br>***Sex/gender:*** *female*	*'Meaning in action'*<br><br>By recognizing and copying the client's meaningful actions, therapists can create brief moments of	*Reappropriated objects*<br><br>Reappropriated objects – e.g. paint-brushed and make-up brushes – can help co-create affective	*Embodied mirroring and musical resonance*<br><br>Mimic the client's bodily actions and expressions – e.g. humming, clapping, foot-tapping –	*Individual talents and abilities*<br><br>The therapists should help autistic individuals find a way of working and living in a way that

	differences in a neuromixed therapeutic context	***Ethnicity/race:*** *NP*<br>***(Clinical) setting:*** *NP*<br>***Additional diagnostic information:*** *Neurodivergent (autism not specified)*	connection, bridging perceptual differences	affordances	to be together in a 'companionable manner'; the other person is not solely a subject of therapeutic intervention	aligns with their talents and abilities<br>*Unique ways of experiencing and understanding the world*<br>The therapist should continuously "self-reflect and be willing to learn" from their client to understand the latter's unique ways of experiencing and understanding the world
Hildebrandt et al. (2016)<br>**Location:**<br>Germany	A randomized controlled trial that examines the effects of a manualized dance and movement therapy program on negative symptoms	***N:*** *78 (treatment group: 55, control group: 23)*<br>***Age:*** *14 – 53 (treatment group: M: 23.07, SD: 8.54, control group: M: 21.27, SD: 5.32)*<br>***Sex/gender:*** *treatment group: 9 female, 44 male, 2 missing, control group: 3 female, 19 male, 1 missing*<br>***Ethnicity/race:*** *majority German*<br>***(Clinical) setting:*** *therapeutic and rehabilitative facilities*<br>***Additional diagnostic information:*** *NP*			*Manualized movement therapy, based on Dance / Movement Therapy*<br>A manualized movement therapy program based on Dance / Movement Therapy, that includes: (1) Chace-Circle, where the therapist imitates the participant's movements to invite engagement; (2) dyadic mirroring, where pairs take turns leading, following, and co-moving; (3) Baum-Circle, where the group mirrors one participant's movement to their chosen music; (4) verbal reflections on feelings and experiences during the sessions	

Jurgens (2023)* <br>**Location:** <br>NA	A theoretical paper that criticizes enactive models for methodological individualism and advocates Chapman's ecological-functional models – framed within a neurodiversity perspective – to better address disability	***N:*** *NA* <br>***Age:*** *NA* <br>***Sex/gender:*** *NA* <br>***Ethnicity/race:*** *NA* <br>***(Clinical) setting:*** *NA* <br>***Additional diagnostic information:*** *NA*	*Focus on the family and social group* <br>By relocation therapeutic attention from the individual to the family or social group, the pressure on autistic individuals to adapt gets reduced and environment adaptations are fostered			*Ecological-functional model*[3] <br>Use the ecological-functional model as an analytical tool to examine disability within the broader organism-environment-ecology context, focusing on relational functioning and reducing stigma by moving away from individual pathology and adaptability
Koch & Kercher (2023) <br>**Location:** <br>Germany	A methodological paper that synthesizes theory and prior empirical findings to present and substantiate the Mirroring Intervention Protocol, which focuses on structured mirroring techniques in dance / movement therapy	***N:*** *feasibility study: 31, RCT: NP.* <br>***Age:*** *NP* <br>***Sex/gender:*** *NP* <br>***Ethnicity/race:*** *NP* <br>***(Clinical) setting:*** *feasibility study: rehabilitation facility, RCT: NP* <br>***Additional diagnostic information:*** *NP*			*Mirroring Intervention Protocol (MIP)* <br>Manualized Dance / Movement Therapy program with four parts (three non-verbal, one verbal) designed to promote kinesthetic empathy, non-verbal skills, body and boundary awareness <br>*Metrics for embodied empathy* <br>Assessment of kinesthetic empathy using a combination of tools: Embodied Intersubjectivity Scale (EIS)[4] – observational items that measure how attuned people feel in joint movement -; Cognitive and Emotional Empathy Questionnaire (CEEQ) (Savage,	

					Dziobek, Teague & Borod 2010, [unpublished manuscript]), movement analysis, and Eberhard-Kächele[5]'s development mirroring taxonomy to identify changes of empathy at the sensorimotor level	
Koehler et al. (2024)<br><br>**Location:**<br><br>Germany	An experimental study that employs machine-learning to build diagnostic classification models, based on quantified reciprocity in naturalistic dyadic conversations between autistic and non-autistic adults	***N:*** *88 (autistic: 28, non-autistic: 60)*<br><br>***Age:*** *18-60 (autistic: M: 37.18, SD: 13.14, non-autistic: M: 31.48, SD: 10.78)*<br><br>***Sex/gender:*** *autistic: 18 female, non-autistic: 26 female*<br><br>***Ethnicity/race:*** *NP*<br><br>***(Clinical) setting:*** *laboratory with video-recorded clinical report*<br><br>***Additional diagnostic information:*** *NA*	*Machine-learning classification based on non-verbal reciprocity*<br><br>A support vector machine learning model was used to differentiate dyads including an autistic person from those with no autistic interaction partner, by quantifying reciprocal adaptation in facial expressions, head motion, and body movements during naturalistic social interactions.		*Dyadic unit of analysis*<br><br>Classification happens at the dyad level, not the individual level	
Ma et al. (2025)<br><br>**Location:**<br><br>China	A qualitative study that employs conversation analysis to examine how therapists manage interactional breakdowns, focusing on pragmatic meaning and multimodal	***N:*** *7*<br><br>***Age:*** *5 – 10 (M:7.7, SD: 1.9)*<br><br>***Sex/gender:*** *2 female, 5 male*<br><br>***Ethnicity/race:*** *NP*<br><br>***(Clinical) setting:*** *Special education*			*Recognizing and harnessing specific modes of interaction*<br><br>Identify pragmatic meanings behind stereotypical actions, such as repeated utterances and rises in prosody vocals, which can foster collaborative	*Avoid ignorance*<br><br>Acknowledge the child's seemingly irrelevant, unrelated utterances as valuable contribution to the interaction; ignoring them can hinder the child's willingness to follow the directive

	resources	*and rehabilitation center of a children's hospital and two autism institutes*<br><br>***Additional diagnostic information:*** *various diagnostic concurrencies: ADHD, non-verbal learning disorder, dysarthria difficulties in executive functions, social interaction, attention*			interaction and intersubjectivity<br><br>*Tailored verbal interactions*<br><br>Tailor verbal instructions to the child's processing abilities, by breaking down complex directives into simples, digestible components<br><br>*Embodied resources*<br><br>Accompany verbal directives with multimodal embodied actions, such as touching, waving, hand clapping, and gestures, to elicit (joint) attention<br><br>*Habitually used stimuli*<br><br>Leverage the child's personal and familiar interest – e.g. special blocks, unique cartoon character – as entry point for the interaction	*Be patient with time gaps*<br><br>Delayed responses require ample time for replies, creating a 'supportive and adaptive environment'
Malcolm et al. (2018)<br><br>**Location:**<br><br>UK	A qualitative study that draws on participant observation and interviews to examine the effects of equine therapy on social behaviors	***N:*** *NP*<br><br>***Age:*** *children*<br><br>***Sex/gender:*** *NP*<br><br>***Ethnicity/race:*** *NP*<br><br>***(Clinical) setting:*** *horse therapy center, small groups from special education schools or units within mainstream*		*Embodied experiences of horse-riding*<br><br>Equine therapy fosters engagement and learning through the embodied, multi-sensory experience of horse-riding<br><br>*Minimized overload*<br><br>Sensory overload can	*'Opening up' the child's world*<br><br>Equine therapy can foster human-human interaction, awareness of self and other, and interspecies intersubjectivity	*Rare sense of mastery*<br><br>Horse-riding offers a rare, empowering experience that allows the rider to 'embody a position of power in a social context'<br><br>*Interspecies interaction 'de-pressurizes'*<br><br>The horse allows for

		schools<br>***Additional diagnostic information:*** *NP*		be prevented by structuring the sensory environment<br>*Paring with horses' personalities*<br>Pairing autistic riders with horses whose temperaments aligns with their preferences can improve comfort and appropriate engagement; while some riders respond well to challenging horses, others benefit from more predictable, stable horses		interaction that is not steered or hindered by social conventions and norms
Maresca et al. (2022)<br>**Location:**<br>NA	A narrative review to examine the impact of hippotherapy on psychological, cognitive, and relational domains in neurodevelopmental disorders, with specific outcomes summarized for autism	***N, aggregated:*** *NP*<br>***Age:*** *pediatric patients*<br>***Sex/gender:*** *NP*<br>***Ethnicity/race:*** *NP*<br>***(Clinical) setting:*** *various international hippotherapy studies*<br>***Additional diagnostic information:*** *NP*	*Immersion in nature and contact with other people*<br>Therapy is supported by sustained sensory and social stimuli<br>*Family involvement*<br>Including parents in therapy may be beneficial for both the client and the family	*Rhythmic sensory-motor input*<br>The horse's repetitive gait offers sensory-motor feedback that leads to improvements in motor and behavior outcomes<br>*Grooming*<br>Close contact with the horse promotes relaxation – e.g. the horse's breathing and sharing its body warmth – fosters psychological health - may be related to the release of oxytocin	*Horse-human interaction to bridge communication gaps*<br>Horses can open as a communication channel between the client and the healthcare professional, fostering the therapeutic alliance	
Martin et al. (2021)	A methodological paper that proposes	***N:*** *NA*	*Immersive environment for*			*Objective measurement*<br>Augment subjective

**Location:**<br>USA	the use of VR with multi-sensor tracking to track quantified behaviors relevant to neuropsychiatric disorders, including autism	***Age:*** *NA*<br>***Sex/gender:*** *NA*<br>***Ethnicity/race:*** *NA*<br>***(Clinical) setting:*** *laboratory immersive environment*<br>***Additional diagnostic information:*** *NA*	*behavior tracking, supporting diagnosis and treatment*<br>Taking the Cave Automatic Virtual Environment[6] as a basis, an immersive environment is suggested that uses quantifiable, objective tools to improve diagnosis of and inform treatment for neuropsychiatric phenomena			reporting from parents, caregivers, and clinicians to support objective, immersive measurements – reducing the risk of bias
Mattson et al. (2022)<br>**Location:**<br>Finland and Norway	A theoretical paper drawing on three Nordic examples of nature-based family therapy to families with children, to foster attuned interaction, emotional bonding, and connectedness to nature	***N:*** *example 1: 55 dyads of fathers and sons, example 2: NP, example 3: NP*<br>***Age:*** *example 1: 10 – 16, example 2: teenagers, example 3: children*<br>***Sex/gender:*** *example 1: male, example 2: female, example 3: male and female*<br>***Ethnicity/race:*** *NP*<br>***(Clinical) setting:*** *mentalization-based family therapy in camps or local nature environments*<br>***Additional diagnostic information:*** *families with autistic children or*	*Nature-based family therapy – 'friluftsterapi' approach*<br>Therapy sessions in outdoor, natural environment to integrate physical activities – e.g. canoeing, hiking, art-making – with therapeutic work can foster communication and social bonding, and provide metaphors to understand and address behavior challenges of the autistic person<br>*Therapy-to-real-world translation*<br>By relocating therapy from the therapy room to daily life natural environments, psychosocial outcomes translate		*Physical empathy*<br>The natural environment allows clients to connect mind and body, making it possible for the therapist to attune to their client's rhythm – fostering, for example, physical empathy	*'Freedom from the role of being a patient'*<br>The natural environment enables a therapeutic relation in which the client is freed from their role as a patient

		children with ADHD. Examples are ‘low-threshold’; no psychiatric diagnoses are needed to participate	better to real-world challenges<br><br>*Nearby nature integration*<br><br>Families can continue activities on their own after therapy, integrating them into daily life thanks to the nearby nature therapy context			
Mazurek et al. (2023)<br><br>**Location:**<br><br>USA	A mixed methods study to examine autistic experiences and preferences with regard to various mental health strategies	***N:*** *303*<br><br>***Age:*** *21 – 71 (M: 37.1, SD: 12.0)*<br><br>***Sex/gender:*** *49.5% cisgender woman, 31.4% cisgender man, 2.0% transgender woman, 4.0% transgender man, 11.6% non-binary/ non-conforming, 1.7% other*<br><br>***Ethnicity/race:*** *5.3% Hispanic/Latino, 0.3% American Indian or Alaska Native, 1.7% Asian, 5% Black or African American, 85.8% White or Caucasian, 6,9% Multiracial*<br><br>***(Clinical) setting:*** *various: cognitive strategies, mindfulness*	*Enhance generalization*<br><br>Focus on the implementation of therapeutic strategies in real-world situations, through in vivo practice[7], homework assignments[8], or in situ technological support[9,10]	*Creative modes of expression*<br><br>Personalized strategies such as ‘pictures’ or ‘creative means to record my story’ can function as an alternative activity to enhance therapy<br><br>*Fidgeting toys*<br><br>Fidgeting toys in the office can function as a personalized strategy		*Therapist acceptance, understanding and validation*<br><br>Acknowledge the needs and feelings of autistic individuals in an informed and non-judgmental way

		meditation, deep breathing, guided imagery or visualization, progressive muscle relaxation, exposure, behavioral activation<br>***Additional diagnostic information:*** *various diagnostic concurrencies: anxiety disorder, depression, ADHD, PTSD, OCD, bipolar disorder*				
Mössler et al. (2023)*<br>**Location:**<br>Norway	An interpretative-hermeneutic study that draws on a video vignette to study the role of physical and institutional affordances on attunement dynamics between an autistic child and non-autistic music therapist	***N:*** *1*<br>***Age:*** *toddler*<br>***Sex/gender:*** *male*<br>***Ethnicity/race:*** *NP*<br>***(Clinical) setting:*** *multi-functional room at primary school*<br>***Additional diagnostic information:*** *NA*		*'Building blocks'*<br>Building blocks are used as a communication channel, which allows the therapist and child to connect without the need for verbal communication<br>*Rearranging the room*<br>Provide an environment that affords joint, creative action	*Responsive musicking*<br>The therapist joins the child's musicking, validating their agency and improving reciprocity	*Idiosyncratic musicking*<br>The therapist acknowledges the idiosyncratic musicking of the child, defying a corrective approach<br>*Institutional flexibility*<br>Institutional changes should support and facilitate atypical, non-normative ways of musicking
Murray et al. (2023)*<br>**Location:**<br>Belgium and	A shared phenomenological self-investigation to explore lived experiences across the autistic/non-autistic divide,	***N:*** *autistic: 3, non-autistic: 1*<br>***Age:*** *NP*<br>***Sex/gender:*** *NP*<br>***Ethnicity/race:*** *NP*			*Interbodily aspects*<br>The autistic context warrants heightened sensitivity to the tangible, interbodily presence of others,	*Bracketing*<br>The use of the phenomenological technique 'bracketing' can help set aside preconceived

UK	focusing on themes such as trust, emotion regulation, and sensory experiences	***(Clinical) setting:*** *monthly 1.5h video conferences*<br>***Additional diagnostic information:*** *NA*			establishing connectedness through mutual adaptation of movement<br><br>*Neuromixed language*<br>A shared use of terminology that alleviates challenges related to ‘translation’ and misinterpretation that often occurs in a clinical context, e.g. ‘sensorium’, ‘social joining’, ‘flocking’	assumptions or theories about each other’s experiences. (Unintentional) ‘fishbowling’ of autism can be prevented, and a mutual understanding and trust can be established<br><br>*Conditions for trust*<br>In neuromixed settings, interaction partners need to be sensitive to differences in power, perspective, and experience, to establish trust<br><br>*Specific states of mind dependent on specific contexts*<br>Traits commonly considered intrinsic to autism – e.g. deficits in empathy or rigid behavior – reflect specific states of mind dependent on specific contexts, reflecting mismatches with socially normative expectations
Nešić et al. (2024)* <br>**Location:**<br>NA	A theoretical-historical analysis that examines the life of Hildegard of Bingen, describing the therapeutic role of medieval monastic environments for	***N:*** *NA*<br>***Age:*** *NA*<br>***Sex/gender:*** *NA*<br>***Ethnicity/race:*** *NA*<br>***(Clinical) setting:*** *the medieval monastic*		*Comfortable socio-material environments*<br>Monastic environments offered structured routines, predictable social interactions, and simple sensory settings that align with autistic		

	autistic individuals	*environment as a socio-material niche*<br>***Additional diagnostic information:*** *NA*		preferences. Modern-day (clinical) settings may mirror these designs to create more attuned therapeutic environments		
Park (2008)<br>**Location:**<br>USA	An ethnographic study that proposes imaginative practices as a means to explore the tension between biomedical and literary-philosophical grammars in medical anthropology	***N:*** *1*<br>***Age:*** *5*<br>***Sex/gender:*** *male*<br>***Ethnicity/race:*** *NP*<br>***(Clinical) setting:*** *a sensory integration-based occupational therapy session in a non-public pediatric clinic*<br>***Additional diagnostic information:*** *NP*		*Enacted narratives and scene-making*<br>Participants engage in scene-making using their bodies, props – e.g. plastic dinosaurs, a therapy ball – and the therapy space in creative, improvisational ways		*Use of literary-philosophical grammars*<br>Therapeutic scene-making draws equally from biomedical and literary-philosophical approaches, both 'integral to a healing of belonging'
Park (2012)<br>**Location:**<br>USA	An ethnographic study drawing on a case study to describe the emergence of embodied metaphors for intersubjectivity in play worlds	***N:*** *5, autistic: 3, ADHD: 2*<br>***Age:*** *5*<br>***Sex/gender:*** *NP*<br>***Ethnicity/race:*** *NP*<br>***(Clinical) setting:*** *play-focused sensory integration clinic, linked with social participation at school*<br>***Additional diagnostic information:*** *NP*		*Embodied metaphors*<br>Objects and gestures in co-created play worlds represent ideas and situations, turning into embodied metaphors of intersubjectivity	*'Throwing breaches'*<br>Deliberate disruptions in the flow of play enable new interactions, renegotiating roles in play and space-sharing	*Laughter and imaginative play*<br>Rather than fixing deficits, moments of shared laughter and imaginative play are viewed as legitimate therapeutic outcomes<br>*Fluid spaces*<br>The physical space, therapeutic materials, and the social structures of the session need to be 'fluid' to enable participants to change

						and evolve their (level of) engagement over time
Rucińska et al. (2021)* <br>**Location:** NP	A theoretical paper drawing on a case study that advances an embodied-enactive account of imagination and metaphor use in autism	***N:*** *1* ***Age:*** *12* ***Sex/gender:*** *male* ***Ethnicity/race:*** *NP* ***(Clinical) setting:*** *a systemic therapy session* ***Additional diagnostic information:*** *NP*		*Embodied imagination and metaphor use* Focus on bodily engagement and sensory experience as a basis for metaphor creation <br>*Physical affordances in metaphor creation* Tailor the environment and provide relevant, physical affordances to support embodied imagination, metaphor creation and use	*Collaborative metaphor creation* Metaphors emerge collaboratively, in interaction, fostering mutual understanding and adjustment	*Challenging linguistic models* Therapeutic work with metaphors should move beyond linguistic models, treating it as a bodily and sensory process
Samaritter & Payne (2017)* <br>**Location:** Netherlands	A qualitative study drawing on video analysis of dance / movement sessions with autistic young adults to identify movements markers of interpersonal attunement	***N:*** *4* ***Age:*** *M: 12.2, SD: 3.8* ***Sex/gender:*** *2 girls, 2 boys* ***Ethnicity/race:*** *NP* ***(Clinical) setting:*** *outpatient child/ youth psychiatric department of a hospital, participants attended special needs education* ***Additional diagnostic information:*** *NP*			*Social Engagement and Attunement (SEAM)* Identification of interpersonal movement markers to measure the success and progress of Dance / Movement therapy	

Samaritter (2017)* <br>**Location:** <br>Netherlands	A qualitative study synthesizing findings from movement observations of dance / movement therapy sessions to develop the *Shared Movement Approach*, which enhances non-verbal interpersonal relating	***N:*** *NP* <br>***Age:*** *NP* <br>***Sex/gender:*** *NP* <br>***Ethnicity/race:*** *NP* <br>***(Clinical) setting:*** *outpatient setting of a mental healthcare institution* <br>***Additional diagnostic information:*** *NP*			*Self-other relations development* <br>Sensorimotor engagement between two persons contribute to self-perception and agency <br>*Shared Movement Approach* <br>Theoretical considerations when engaging in Dance / Movement Therapy, such as the procedural structure of the setting, relational modes – mirroring, responding to the child's movement, etc. – and movement actions – spontaneous responses, contrasting movements, etc.
Shaughnessy et al. (2024) <br>**Location:** <br>UK	A qualitative study drawing on interviews and qualitative thematic analysis to examine how music can support everyday functioning, care routines, and wellbeing in autistic children	***N:*** *25* <br>***Age:*** *4 – 9 (M: 5.8)* <br>***Sex/gender:*** *23 male, 2 female* <br>***Ethnicity/race:*** *of participating families: n: 4 White background, 7 mixed ethnic backgrounds, 5 Asian ethnic backgrounds, 4 Black ethnic backgrounds, 5 any other ethnic*	*Music in everyday routines* <br>Music is integrated into everyday activities <br>*Scaffolding everyday routines* <br>Music as a 'regulating tool' can help transition changes in 'mood and energy', provide a sensory release when frustrated or help express frustration	*Create a comfortable environment* <br>*Parents described that, when children were given a safe and inviting space to play – both creatively and socially – mutual interaction flourished*	*Music to support communication* <br>Predominantly non-verbal children can be supported in finding an alternative way to express themselves - with more clarity and confidence <br>*Shared musical play* <br>Musical play scaffolds interactivity as it invites caregivers and autistic

		background<br>***(Clinical) setting:*** *musical home contexts, n: 4 parents play an instrument, 21 none*<br>***Additional diagnostic information:*** *verbal abilities: n: 4 no language, n: 10 signs/single words, n: 7 simple sentences, n: 4 full sentences*	through music, as well as provide a sense of fun in the development of everyday skills, for example by making up and singing a song when teeth brushing<br>*Empower parents with musicality*<br>Music practitioners, therapists, and educators can empower parents by equipping them with practical musical resources and strategies that can be easily applied at home		to play with each other. The 'musical dialogue' makes the musical play bidirectional. Here, musical play allows the children to explore their own musical worlds and imagined narratives	
Solomon (2015)<br>**Location:**<br>USA	A qualitative paper drawing on two case studies that examines 'being social' for autistic children across two settings: a psychological interview in a mental health clinic and in animal-assisted activities	***N:*** *2*<br>***Age:*** *9*<br>***Sex/gender:*** *female*<br>***Ethnicity/race:*** *NP*<br>***(Clinical) setting:*** *urban mental health clinic and an animal-assisted activity session including a therapy dog and therapy cat*<br>***Additional diagnostic information:*** *PDD-NOS and Autistic Disorder*		*Use of companion animals*<br>Companion animals mediate experiences of intersubjectivity that are not afforded in human-only interactions, creating an 'interactional substrate' that shows signs of sociality, intersubjectivity, and affect at the side of the autistic child		*Challenging human-centered therapeutic views*<br>The inclusion of animals shifts the therapeutic focus beyond human-only interaction

Stallman et al. (2022)<br><br>**Location:**<br><br>NP	A methodological paper that designed and proposes a classroom VR environment to study social emotion regulation via an embodied virtual agent	***N:*** *autistic: 4, overall: 20 (pilot, ongoing at the time of publication)*<br><br>***Age:*** *overall: 9 – 17*<br><br>***Sex/gender:*** *NP*<br><br>***Ethnicity/race:*** *NP*<br><br>***(Clinical) setting:*** *VR lab and off-site VR setups*<br><br>***Additional diagnostic information:*** *NP*	*Virtual Reality (VR) for exploring emotion regulation and social support*<br><br>VR can create controlled, immersive environments with virtual agents to study and train emotion regulation strategies, focusing on the type and impact of peer support (reappraisal, distraction, or presence only)			
Taels et al. (2023)<br><br>**Location:**<br><br>Belgium	An interpretative-phenomenological analysis drawing on interviews with autistic individuals about their experiences of hypersensitivity, focusing on bodily self-experience, perception of time and space, sense-making, intersubjectivity, and mood	***N:*** *18*<br><br>***Age:*** *22 - 65*<br><br>***Sex/gender:*** *13 female, 5 male*<br><br>***Ethnicity/race:*** *NP*<br><br>***(Clinical) setting:*** *No participant was hospitalized at the time of the interview*<br><br>***Additional diagnostic information:*** *ASD: 10, Autistic Disorder: 3, Asperger Disorder: 5, 8 participants with past psychiatric hospitalization*		*Stimulating practice and activity*<br><br>Activities that involve physical movement may ‘counteract a fragile sense of embodiment’<br><br>*Predictable sensory input*<br><br>Social experiences are more manageable if autistic individuals have a feeling of control over the sensory environment	*Taking initiative*<br><br>Social interaction and physical proximity is easier if autistic individuals are the ones who initiate it	
Turowetz & Maynard	An ethnographic study that employs	***N:*** *49*	*Diagnosis as an embodied, interactive*			*Interaction order of the clinic*[11]

(2019)<br><br>**Location:**<br><br>USA	conversation analysis to examine how clinicians conduct autism diagnostic assessments – using the ADOS as an exemplar -, highlighting how the interactional context is obscured	***Age:*** *children*<br><br>***Sex/gender:*** *NP*<br><br>***Ethnicity/race:*** *NP*<br><br>***(Clinical) setting:*** *'Central Developmental Disabilities Clinic' (pseudonym), specialized in the diagnosis of childhood developmental disorders*<br><br>***Additional diagnostic information:*** *NP*	*process*<br><br>Diagnosis happens in an interactional and material context; in interaction with a clinician, the physical room and its artefacts – embodied, interactional aspects that are obscured in standardized assessment protocols			Provide a report on the social and procedural underpinnings of the diagnosis assessment, including how tests were conducted, how judgements were made, how diagnoses are determined and communicated to parents<br><br>*Engage with instantiation stories for treatment recommendations*[12]<br><br>By focusing on specific moments when a child interacts with others, treatment can be personalized - using rich, real-life information of how the child behaves and makes sense of the world.
Vaisvaser (2024)*<br><br>**Location:**<br><br>NA	A theoretical paper at the intersection of neuroscience, phenomenology, and psychotherapy that conceptualizes Creative Arts Therapies as fostering, shaping, and integrating selfhood	***N:*** *NA*<br><br>***Age:*** *NA*<br><br>***Sex/gender:*** *NA*<br><br>***Ethnicity/race:*** *NA*<br><br>***(Clinical) setting:*** *NA*<br><br>***Additional diagnostic information:*** *NA*		*Creative Arts Therapies (CATs) for self-other differentiation and selfhood*<br><br>By allowing clients a safe and predictable space for aesthetic and artistic exploration, CATs (Dance / Movement Therapy, art therapy, music therapy, drama therapy, psychodrama, and bibliotherapy) foster multiple nested, self-related processes that help with self-other differentiation and self-	*Rhythmic synchronization between therapist and client - entrainment*<br><br>Neural intersubjective synchrony can emerge from behavioral synchrony during therapeutic interactions, which fosters shared meaning, intentionality, and interpretation	

				cultivation		
Van Huizen et al. (2024)*<br>**Location:**<br>Netherlands	A theoretical paper drawing on co-design case studies to propose an integrated framework for incorporating lived experiences in supportive technology design	***N:*** *11+ (10 case studies with one person, 1 case study with 'multiple participants')*<br>***Age:*** *14 – 39*<br>***Sex/gender:*** *NP*<br>***Ethnicity/race:*** *NP*<br>***(Clinical) setting:*** *9 participants joined from mental healthcare organizations*<br>***Additional diagnostic information:*** *NP*	*Habitual experience in supportive technology design*<br>Consider how the supportive technology aligns with pre-existing habits and routines	*Sensory experience in supportive technology design*<br>Consider how the supportive technology aligns with sensory preferences	*Social experience in supportive technology design*<br>Consider how the supportive technology enables an end user to communicate in their own preferred ways	*Overcome a deficit-centered frame in supportive technology design*<br>Move beyond deficit-centered frames in supportive technology design; instead, prioritize supportive integration into autistic lifeworlds<br>*Societal experience in supportive technology design*<br>Consider how the supportive technology consolidates or otherwise challenges socio-cultural images of autism<br>*Participatory design*<br>End users should be active partners in the design process, to ensure their lived experiences are well-reflected in the supportive technology
Vulcan (2016)<br>**Location:**<br>Israel	A qualitative paper drawing on interviews to study the lived experiences of therapists working with autistic children, highlighting the body's role in	***N:*** *28 practicing therapists*<br>***Age:*** *NP*<br>***Sex/gender:*** *24 female, 4 male*<br>***Ethnicity/race:*** *NP*<br>***(Clinical) setting:***			*'Bodily listening'*<br>Bodily engagement offers an alternative way of communicating when verbal communication is not tenable	

	intersubjectivity	*various: social work, clinical psychology, music therapy, drama therapy, dance / movement therapy, special education kindergartens, primary, and secondary schools*<br><br>***Additional diagnostic information:*** *practices with autistic children aged between 2 and 16, no difference between high- and low-functioning clients*			*'From body to body'*<br><br>Physical, bodily sensations at the side of the therapist can be indicative of (preconscious) mental experiences – 'somatic empathy'<br><br>*Implicit bodily relational knowing*<br><br>To meet the autistic child on their (non-verbal) level, therapist can connect with the basic parts of themselves to achieve intersubjective engagement with the child, using their own bodily sensations, early memories, and preverbal experiences – 'tapping into embodied rather than cognitive ways of knowing'	
Xavier et al. (2023)<br><br>**Location:**<br><br>NA	A theoretical paper that proposes that morphological, behavioral, and motor similarities between autistic children and peers can foster the development of self-consciousness through imitative actions	***N:*** *NA*<br><br>***Age:*** *children*<br><br>***Sex/gender:*** *NA*<br><br>***Ethnicity/race:*** *NA*<br><br>***(Clinical) setting:*** *NA*<br><br>***Additional diagnostic information:*** *NA*			*Peer-mediated interventions (PMI)*<br><br>Morphological, behavioral, and motor similarities between autistic children might make it easier to engage in motor imitation – a 'situation of interpersonal affordance' – which helps to foster a sense of self and self-other differentiation. Targets for PMI are Dance / Movement Therapy and naturalistic	

	imitation intervention[13]

Cited work

1. Sparrow SS, Cicchetti DV, Saulnier C. *Vineland-3: Vineland Adaptive Behavior Scales*. 3rd ed. PsychCorp; 2016.
2. Cook WL, Kenny DA. The Actor–Partner Interdependence Model: A model of bidirectional effects in developmental studies. *Int J Behav Dev*. 2005;29(2):101-109. doi:10.1080/01650250444000405
3. Chapman R. Neurodiversity and the Social Ecology of Mental Functions. *Perspect Psychol Sci*. 2021;16(6):1360-1372. doi:10.1177/1745691620959833
4. Fuchs T, Koch SC. Embodied affectivity: on moving and being moved. *Front Psychol*. 2014;5:508. doi:10.3389/fpsyg.2014.00508
5. Eberhard-Kächele M. Spiegelungsphänomene in der Tanztherapie/Körperpsychotherapie. In Bender S, ed. *Bewegungsanalyse von Interaktion – Movement Analysis of Interaction*. Logos Verlag; 2010:193-212
6. Cruz-Neira C, Sandin DJ, Defanti TA. Surround-screen projection-based virtual reality: the design and implementation of the CAVE. *Proc 20th Annu Conf Comput Graph Interact Tech SIGGRAPH 93*. Association for Computer Machinery. 1993:135-142. doi: 10.1145/166117.166134
7. Abramowitz JS, Arch JJ. Strategies for Improving Long-Term Outcomes in Cognitive Behavioral Therapy for Obsessive-Compulsive Disorder: Insights From Learning Theory. *Cogn Behav Pract*. 2014;21(1):20-31. doi:10.1016/j.cbpra.2013.06.004
8. Kazantzis N, Deane FP, Ronan KR. Homework assignments in cognitive and behavioral therapy: A meta-analysis. *Clin Psychol Sci Pract*. 2000;7(2):189-202. doi:10.1093/clipsy.7.2.189
9. Loo Gee B, Griffiths KM, Gulliver A. Effectiveness of mobile technologies delivering Ecological Momentary Interventions for stress and anxiety: a systematic review. *J Am Med Inform Assoc*. 2016;23(1):221-229. doi:10.1093/jamia/ocv043
10. Swan AJ, Carper MM, Kendall PC. In Pursuit of Generalization: An Updated Review. *Behav Ther*. 2016;47(5):733-746. doi:10.1016/j.beth.2015.11.006
11. Maynard DW, Turowetz J. Doing Abstraction: Autism, Diagnosis, and Social Theory. *Sociol Ther*. 2019;37(1):89-116. doi:10.1177/0735275119830450
12. Maynard DW, Turowetz J. Doing Diagnosis: Autism, Interaction Order, and the Use of Narrative in Clinical Talk. *Soc Psychol Q*. 2017;80(3):254-275. doi:10.1177/0190272517720683
13. Ingersoll B. The Social Role of Imitation in Autism: Implications for the Treatment of Imitation Deficits. *Infants Young Child*. 2008;21(2):107-119. doi:10.1097/01.IYC.0000314482.24087.14
